# Correlations between SS-OCT and OCT angiography biomarkers in treatment-naïve neovascular AMD during aflibercept therapy: a prospective study

**DOI:** 10.1186/s40942-026-00854-x

**Published:** 2026-04-28

**Authors:** Marcussi Palata Rezende, Fernanda Atoui Faria, Daniel Prado Beraldo, Julia Polido, Rubens Belfort Jr, Thiago Cabral

**Affiliations:** 1 Department of Ophthalmology, Federal University of São Paulo (UNIFESP), São Paulo, SP Brazil; 2 Clinica Oftalmo-Retina, Presidente Prudente, SP Brazil; 3 Department of Ophthalmology, Federal University of Espírito Santo (UFES), Vitória, ES Brazil

**Keywords:** Neovascular age-related macular degeneration, Aflibercept, Biomarkers, Swept-source OCT, OCT angiography, Macular thickness, Choroidal thickness, Superficial plexus avascular area, Deep plexus avascular area, Macular neovascularization area

## Abstract

**Background:**

Swept-source OCT (SS-OCT) and OCT angiography (SS-OCTA) enable high-resolution assessment of retinal and choroidal biomarkers in neovascular AMD (nAMD). However, prospective analyses and how these biomarkers correlate before and after therapy are limited. The aim of this study was to prospectively evaluate biomarker correlations following a loading dose of aflibercept using state-of-the-art, high-resolution imaging with SS-OCT and SS-OCTA over the course of a 4-month follow-up in treatment-naïve nAMD.

**Methods:**

This prospective interventional case series study included 21 eyes from 21 treatment-naïve patients with nAMD. All eyes received three monthly intravitreal aflibercept injections. Patients were evaluated at baseline and one month after the loading phase (4-month visit). The biomarkers included the best-corrected visual acuity (BCVA), central macular thickness (CMT), central choroidal thickness (CCT), macular neovascularization area (MNVA), vessel density (VD), and avascular area of the superficial plexus (AASP) and deep plexus (AADP). Pre (baseline) and posttreatment values were compared, and correlations were analyzed using Pearson’s or Spearman’s methods.

**Results:**

Significant functional, structural, and vascular improvements in all the evaluated biomarkers except intraocular pressure were observed after treatment. The mean BCVA improved from 1.11 ± 0.47 to 0.56 ± 0.20 logMAR (*p* < 0.001). SS-OCT (CMT and CCT), and SS-OCTA (MNVA, AASP and AADP) metrics were significantly lower following treatment. Correlation analysis revealed multiple statistically significant associations among structural, functional, and vascular biomarkers. The baseline CMT showed a moderate positive correlation with the CMT posttreatment (*r* = 0.53; *p* = 0.013) and a very high negative correlation with CMT reduction (*r* = − 0.95; *p* < 0.001). The baseline CCT was highly correlated with CCT posttreatment (*r* = 0.87; *p* = 0.002). The BCVA correlated significantly with CMT at baseline and posttreatment. Notably, the baseline MNVA was moderately negatively correlated with the posttreatment CCT (*r* = − 0.51; *p* = 0.019). Significant correlations were also identified between the AADP and both the baseline CMT and CMT reduction, as well as between changes in AASP and CCT. These findings highlight complex and interdependent relationships among retinal thickness, choroidal structure, visual function, and OCTA-derived microvascular biomarkers following aflibercept therapy.

**Conclusions:**

In treatment-naïve eyes with nAMD, aflibercept loading therapy induces significant and coordinated functional, structural, and microvascular changes. The observed correlations demonstrate that baseline SS-OCT and SS-OCTA biomarkers not only reflect initial disease burden but are also closely associated with the magnitude and direction of anatomical and vascular remodeling after treatment. These findings support the value of integrated multimodal biomarker assessment for improving prognostic stratification and advancing individualized management strategies in treatment-naïve nAMD. These findings are novel and have not been previously reported in the literature.

**Clinical trial number:**

Not applicable.

## Background

Biomarkers provide objective, quantifiable measures of biological processes, disease activity, and treatment response, enabling earlier detection, risk stratification, and monitoring of disease trajectories. In clinical research, validated biomarkers can serve as intermediate endpoints that improve trial efficiency and support prognostic assessment and a more personalized, evidence-based approach to care [1].

During both initial evaluation and treatment follow-up for patients with neovascular age-related macular degeneration (nAMD), optical coherence tomography (OCT) is often used to predict and evaluate treatment response and guide treatment [2]. When Swept-source optical coherence tomography (SS-OCT) are paired in swept-source optical coherence tomography angiography (SS-OCTA), these advantages are enhanced, offering more rapid and detailed imaging of deeper vascular layers and providing comprehensive structural and vascular assessments within a single examination [3, 4].

Structural and vascular biomarkers obtained through OCT and OCTA provide complementary insights into disease activity in patients with nAMD. Structural parameters, such as the central macular thickness (CMT) and central choroidal thickness (CCT), intraretinal fluid (IRF) and subretinal fluid (SRF), and pigment epithelial detachment (PED), offer objective views of retinal injury and reflect key biological processes driving vision decline. Moreover, OCTA provides a vascular perspective, allowing noninvasive assessment of vessel density (VD), Macular neovascularization (MNV) morphology, and alterations within the superficial and deep avascular plexuses. Together, these imaging biomarkers deepen our understanding of how the retina and choroid respond to anti-vascular endothelial growth factor.

(anti-VEGF) therapy, helping clinicians interpret anatomical and microvascular changes more precisely and supporting increasingly individualized care [5]. Furthermore, several investigations have explored whether quantitative biomarkers derived from OCT and OCTA can improve the prognosis of nAMD by characterizing the MNV itself. Machine learning models applied to large, standardized OCT datasets have demonstrated that Three-dimensional (3D) morphological descriptors of IRF, SRF, and PED have prognostic value and can improve the prediction of functional outcomes during anti-VEGF therapy, particularly when early treatment phase data are incorporated [6]. Other studies have shown that specific neovascular complex features, including the vessel area, total vessel length, total number of junctions, and junction density, respond dynamically to anti-VEGF and, in some cases, correlate significantly with retinal thickness measurements, suggesting structural and vascular coupling within the MNV [7]. Additional studies using quantitative OCT- and OCTA-derived metrics have shown that specific baseline morphological and qualitative characteristics of MNV are associated with visual outcomes and treatment response following anti-VEGF therapy, underscoring the clinical relevance of detailed morphologic profiling [8]. Moreover, the CMT has been correlated with vascular OCTA parameters, reinforcing that distinct biomarkers may reflect neovascular activity and treatment response across imaging modalities [9]. Nevertheless, despite these advances, substantial variability in individual treatment outcomes persists across studies, underscoring ongoing controversies regarding the predictive accuracy of currently available biomarkers and reinforcing the need for further research and the identification of additional, more robust structural and vascular indicators of therapeutic response in patients with nAMD [9].

Several studies have investigated OCT and OCTA-based biomarkers, but studies that systematically explore their correlations throughout treatment remain scarce, especially studies of treatment-naïve patients. Furthermore, prospective evidence on how retinal and choroidal metrics interact during anti-VEGF therapy in patients with nAMD is lacking. Thus, identifying how different biomarkers correlate with one another in the case of a disease that can be actively treated is particularly relevant. This understanding forms the central objective of our study.

To our knowledge, this is the first prospective study to examine how retinal and choroidal biomarkers derived from SS-OCT and SS-OCTA correlate before and after a loading dose of aflibercept (three monthly injections) in naïve patients with nAMD. By comprehensively analyzing these parameters, we elucidated the structural and vascular changes that accompany the therapeutic response. We applied state-of-the-art imaging technology, and our work contributes to improving our understanding by exploring how these biomarkers behave together throughout treatment. Ultimately, we hope that these findings will help promote a more individualized approach to the treatment of nAMD, offering new insights into how aflibercept therapy influences retinal and choroidal microenvironments and highlighting the potential of SS-OCT and SS-OCTA biomarkers to guide more personalized management. Thus, the aim of this study was to evaluate treatment-naïve patients with nAMD using SS-OCT and SS-OCTA at baseline and at posttreatment, defined as one month after completion of a standard 3-dose aflibercept loading regimen, corresponding to a total follow-up period of 4 months. The primary objectives were as follows: (1) to assess whether statistically significant correlations exist among key functional, structural, and vascular biomarkers measured at baseline and post-treatment, including best-corrected visual acuity (BCVA), CMT, CCT, macular neovascularization area (MNVA), avascular area of the superficial plexus (AASP), avascular area of the deep plexus (AADP), and VD. And (2) to evaluate changes in choroidal and macular structural and vascular biomarkers following treatment.

## Methods

### Study design and population

Participants were recruited at Clínica Oftalmo-Retina, Presidente Prudente, São Paulo, Brazil. The investigation followed a prospective, noncomparative interventional case-series design. Eligibility required a diagnosis of nAMD and the absence of any previous treatment for the condition.

A) **Inclusion criteria**:

Patients were eligible for the study if they met the following conditions:

1. Adults ≥ 50 years diagnosed with unilateral nAMD characterized by type 1 MNV and who were treatment-naïve for anti-VEGF therapy were included.
2. Loading injections were delivered by a single ophthalmologist throughout the study period.
3. Comprehensive records, including demographics, current medications, intraocular pressure (IOP), BCVA, and SS-OCT/SS-OCTA imaging before and after therapy, were available.
4. BCVA within 20/25 to 20/400 was obtained using the Early Treatment Diabetic Retinopathy Study (ETDRS) protocol and is expressed as Snellen values.
5. Signed informed consent documents were obtained from all participants and stored in the patient charts.

This restriction to unilateral disease eliminated potential inter-eye correlation bias.

B) **Exclusion criteria**:

Patients were excluded if any of the following applied:

1. The patient received any prior intravitreal anti-VEGF therapy.
2. The patient had an incomplete loading phase, which was defined as the failure to receive all three scheduled intravitreal injections for any cause.
3. The patient had any coexisting retinal or optic nerve disease that could confound the outcomes, including diabetic retinopathy, glaucoma, vitreoretinal interface disorders (epiretinal membrane, macular hole, proliferative vitreomaculopathy) and inherited retinal dystrophies.
4. The patient had a history of ocular surgery, such as retinal detachment repair or glaucoma procedures, which is likely to affect macular status or IOP control.

The prospective design was central for biomarker identification because it allowed standardized visit timing (baseline and after the aflibercept loading phase, 4 months) and a uniform SS-OCT/SS-OCTA acquisition and quality control workflow, reducing measurement variability and information bias. Consecutive enrollment of treatment-naïve eyes before outcome assessment minimizes selection bias and preserves the temporal sequence from baseline phenotype to early treatment response, improving internal validity for exploratory, hypothesis-generating biomarker analyses [10, 11, 12].

A control group was not included given the study’s purpose and ethical constraints. The project followed a prospective interventional design, in which retinal biomarkers in patients with nAMD were measured at baseline and after a standardized loading regimen. Creating an arm without treatment simply for comparison would deny participants access to therapy regarded as standard care in this setting and would contradict the principles outlined in the Declaration of Helsinki. Therefore, a no-treatment control arm would have been ethically unjustifiable in this context. By using each eye as its own comparator across time, the internal validity of the study for the targeted outcomes was preserved. Furthermore, the study met the ethical requirements to treat eligible patients.

### Ethical committee approval

This research followed Resolution 196/96 of the National Health Council (Ministry of Health, Brazil) and was conducted in accordance with the principles of the Declaration of Helsinki. The study protocol underwent ethical review and received formal approval from the Ethics Committee of the Hospital Regional do Câncer da Santa Casa de Misericórdia de Presidente Prudente, São Paulo, under CAAE: 19386619.1.0000.8247.

### Data collection and examination techniques

From September 2019 to September 2022, individuals who fulfilled the eligibility requirements and provided written consent were prospectively included. Once nAMD was confirmed and the core dataset was obtained, assessments covered demographic age, sex, systemic and ocular antecedents and a comprehensive ophthalmologic examination. At baseline (pretreatment), the protocol included collecting the patient’s medical history, BCVA, and IOP and performing slit-lamp biomicroscopy, a dilated fundus examination, seven-field color photography, red-free imaging, fundus autofluorescence imaging, fluorescein angiography, SS-OCT, and SS-OCTA. The entire panel of tests was repeated four weeks after the loading phase was completed. The BCVA was obtained using Snellen charts and converted to the logarithm of the minimum angle of resolution (logMAR) for statistical analysis.

Retinal and choroidal imaging was performed with SS-OCT and SS-OCTA on a DRI-OCT Triton system (Topcon, Tokyo, Japan). Automated layer segmentation was applied, and the results were subsequently reviewed. In SS-OCT, 7 × 7 mm cubes were used; in SS-OCTA, 4.5 × 4.5 mm scans were used. Retinal thickness was defined as the distance from the vitreoretinal interface to the RPE. Choroidal thickness was defined as the distance from the border of the RPE to the chorio-scleral boundary. The device’s calibration tools provided these distance measurements. The CMT and CCT were defined as the mean thickness within the central 1000 μm of the ETDRS grid using the macular and choroidal maps provided by the SS-OCT software. Each segmentation line, namely, the vitreoretinal interface, Retinal pigment epithelium (RPE), and chorio-scleral boundary, was inspected and manually corrected when needed. SS-OCTA outputs included the following: (A) VD was calculated automatically by the device software (IMAGEnet 6) as a global retinal vessel density metric, incorporating both superficial capillary plexuses (SCP) and deep capillary plexuses DCP, as per manufacturer definition; (B) Foveal Avascular Zone (FAZ) in the superficial and deep layers, recorded as AASP and AADP, respectively; (C) MNVA. These areas (B and C) were traced manually by an experienced ophthalmologist and verified by a second reader because the software does not generate them automatically. Image acquisition and grading were masked. Two independent ophthalmologists analyzed all the scans (SS-OCT and SS-OCTA), and disagreements were adjudicated by a third reviewer.

A trained imaging technician, who was not aware of either the study procedures or each patient’s treatment status, performed all the SS-OCT and SS-OCTA scans. Examinations were systematically conducted at approximately 10:00 a.m. to mitigate diurnal changes in choroidal thickness, which can affect quantitative measurements [13, 14].

The initial step involved a loading regimen of aflibercept administered intravitreally once per month for three consecutive months (loading dose). The second step was a postloading evaluation performed one month after the third injection; thus, the outcomes were analyzed approximately four months after the baseline data were collected. Imaging with SS-OCT and SS-OCTA was performed at baseline and repeated one month after the third injection to quantify anatomical and microvascular changes. All procedures were carried out by a single senior ophthalmologist in a surgical suite. After completion of the loading dose, participants continued under surveillance and received further treatment if signs of nAMD remained. Injections were standardized as follows: aflibercept 0.05 ml 2 mg; concentration 40 mg/ml; injected via pars plana in the superotemporal quadrant; 30-gauge needle 0.3 × 13 mm; and entry site 3.5 mm posterior to the limbus.

A correlation is a statistical tool used to evaluate whether two continuous variables move together and, if so, in what direction and with what intensity. As explained by Mukaka (2012),^15^ the correlation coefficient (r) is a dimensionless value ranging from − 1 to + 1, where − 1 represents a perfect negative relationship, + 1 represents a perfect positive relationship, and 0 indicates no linear association. The correlation strength can be interpreted following a widely accepted approach: values between 0.00 and 0.19 suggest a negligible relationship; values between 0.20 and 0.39 suggest a low relationship; values between 0.40 and 0.59 suggest a moderate relationship; values between 0.60 and 0.79 suggest a high relationship; and values between 0.80 and 1.00 suggest a very high correlation (Table 1) [15]. In clinical practice, these insights support more individualized decision-making, help refine treatment strategies and help anticipate therapeutic responses, ultimately improving the care of complex diseases [15, 16].

**Table 1:** Rule of thumb for interpreting the size of a correlation coefficient

Size of Correlation	Interpretation
0.90 to 1.00 (− 0.90 to − 1.00)	Very high positive (negative) correlation
0.70 to 0.90 (− 0.70 to − 0.90)	High positive (negative) correlation
0.50 to 0.70 (− 0.50 to − 0.70)	Moderate positive (negative) correlation
0.30 to 0.50 (− 0.30 to − 0.50)	Low positive (negative) correlation
0.00 to 0.30 (0.00 to − 0.30)	Negligible correlation

Adapted from Mukaka MM. Statistics Corner: A guide to appropriate use of correlation coefficient in medical research. Malawi Med J. 2012 Sep;24(3):69–71

### Statistical analysis

Correlations: A Gaussian distribution test was performed to verify a normal distribution in the groups analyzed. Correlation analyses between continuous variables were performed using Pearson’s correlation when the groups presented a normal distribution and Spearman’s correlation for groups without a normal distribution. In addition, 95% confidence intervals for the correlation coefficients were calculated using Fisher’s z-transformation. Correlation analyses were predefined based on biological plausibility between structural retinal parameters, choroidal measurements, and OCTA-derived vascular biomarkers. Because the study was designed as an exploratory biomarker investigation, no formal adjustment for multiple comparisons was applied, and results should therefore be interpreted as hypothesis-generating.

Pre- (baseline) and posttreatment comparisons: were comparisons were performed using paired statistical tests, as measurements were obtained from the same eyes. The normality of the differences between pre- and post-treatment values was assessed using the Kolmogorov–Smirnov test. For variables with approximately normally distributed differences, the paired Student’s t-test was applied. When normality was not met, the Wilcoxon signed-rank test was used.

Patient characteristics: The population characteristics, such as age and sex, are presented in tables with mean and standard deviation measurements.

To perform the statistical analysis, IBM SPSS v.24 software was used, and a significance level of 5% was adopted for all analyses. Given the exploratory nature of this study, adjustments for multiple comparisons were not applied, in line with the study objective of hypothesis generation rather than confirmatory inference.

## Results

### General results

A total of 21 eyes from 21 treatment‑naïve patients diagnosed with nAMD were included. The mean age of the patients was 79.76 ± 9.13 years. Women represented 52.39% of the cohort (*n* = 11), and men represented 47.61% (*n* = 10). The studied eye was the right eye in 38.10% of the patients (*n* = 8) and the left eye in 61.90% of the patients (*n* = 13). With respect to lens status, 23.80% of the eyes were phakic (*n* = 5), whereas 76.20% were pseudophakic (*n* = 16), as summarized in Table 2. Baseline demographic and clinical characteristics of the study population.

**Table 2:** Description of the clinical and personal characteristics of the participants

Variable	Value
Number of patients	21
Number of eyes	21
Age, years (mean ± SD)	79.76 ± 9.13
Gender, n (%)	
Men	10 (47.61%)
Women	11 (52.39%)
Eye, n (%)	
Right	8 (38.10%)
Left	13 (61.90%)
Lens status, n (%)	
Phakic	5 (23.80%)
Pseudophakic	16 (76.20%)

Significant anatomical and functional improvements in all biomarkers were observed after three monthly intravitreal aflibercept injections, except for the IOP, which remained unchanged (Table 3. Description of pre and post intervention groups). The BCVA improved from 1.11 ± 0.47 to 0.56 ± 0.20 logMAR (*p* < 0.000). The CMT decreased from 340.90 ± 108.38 μm at baseline to 219.14 ± 34.68 μm (*p* < 0.000). The CCT also significantly decreased, from 172.19 ± 78.58 μm to 138.62 ± 79.80 μm (*p* < 0.000). The IOP remained stable (12.00 ± 1.05 mmHg vs. 12.09 ± 1.26 mmHg, *p* = 0.602). The SS-OCTA metrics also demonstrated significant changes. The MNVA decreased from 2,397.97 ± 1,359.73 mm² to 1,128.03 ± 890.45 mm² (*p* < 0.000). The VD also decreased significantly, from 26.11 ± 13.69% to 18.36 ± 6.78% (*p* = 0.017). The AASP significantly decreased (310.51 ± 158.97 mm² to 260.15 ± 134.54 mm², *p* = 0.042), and the AADP significantly decreased from 1,559.24 ± 1,707.52 mm² to 368.79 ± 154.92 mm² (*p* < 0.000).

**Table 3:** Description of pre and post intervention groups

Variable	Pre	Post	*P*-value
BCVA (logMAR)	1.11 ± 0.47	0.56 ± 0.20	< 0.001
CMT (µm)	340.90 ± 108.38	219.14 ± 34.68	< 0.001
CCT (µm)	172.19 ± 78.58	138.62 ± 79.80	< 0.001
IOP (mmHg)	12 ± 1.05	12.09 ± 1.26	0.602
MNVA (mm^2^)	2,397.97 ± 1,359.73	1,128.03 ± 890.45	< 0.001
VD	26.11 ± 13.69	18.36 ± 6.78	0.017
AASP (mm^2^)	310.51 ± 158.97	260.15 ± 134.54	0.042
AADP (mm^2^)	1,559.24 ± 1,707.52	368.79 ± 154.92	< 0.001

P-values were calculated using paired statistical tests. The paired Student’s t-test was applied for variables with approximately normally distributed differences, and the Wilcoxon signed-rank test was used when normality was not met. Values less than 0.05 indicate statistical significance

BCVA: best-corrected visual acuity; logMAR: logarithm of the minimum angle of resolution; CMT: central macular thickness; CCT: central choroidal thickness; IOP: intraocular pressure; MNVA: macular neovascularization area; VD: vessel density; AASP: avascular area of the superficial plexus; AADP: avascular area of the deep plexus

**Fig. 1:**
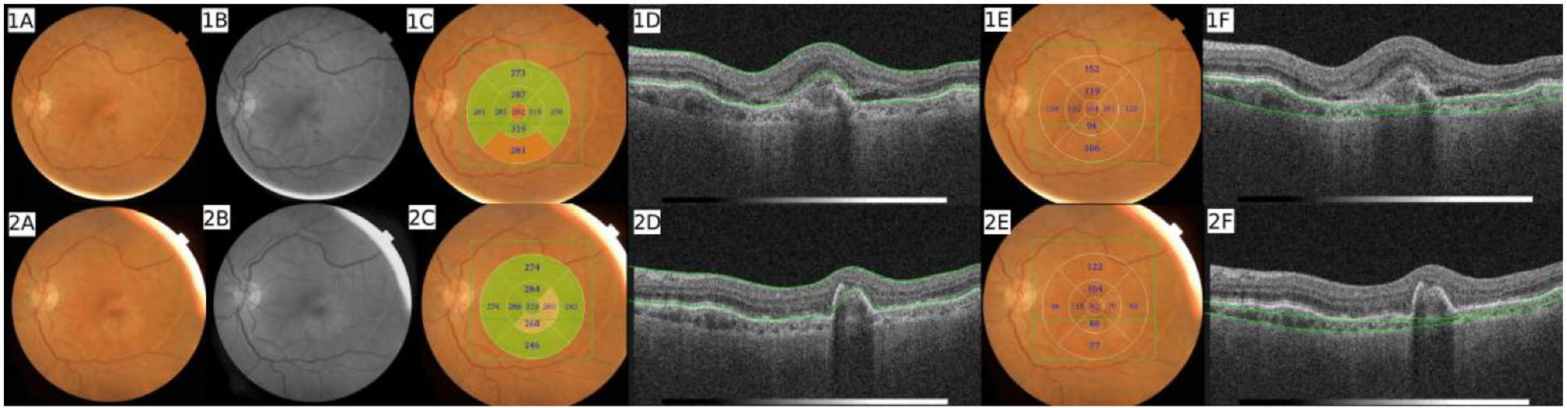
Images labeled with 1 correspond to the baseline evaluation, whereas images labeled with 2 correspond to the posttreatment evaluation (one month after the third aflibercept injection). (**1A**) Color fundus photograph demonstrating retinal pigment epithelium (RPE) rarefaction and the presence of drusen. (**1B**) Red-free fundus image. (**1C**) Pretreatment macular thickness map showing increased central macular thickness (CMT = 282 μm), with a subsequent reduction in the posttreatment map (**2C**) (CMT = 229 μm). (**1D**) Pretreatment SS-OCT B-scan illustrating macular thickness segmentation, intraretinal and subretinal fluid, macular neovascular membrane, and pigment epithelial detachment (PED). (**2D**) Posttreatment SS-OCT B-scan demonstrating macular thickness segmentation with complete resolution of intraretinal and subretinal fluid. (**1E**) Pretreatment choroidal thickness map showing central choroidal thickness (CCT = 104 μm), with a reduction observed posttreatment (**2E**) (CCT = 82 μm). (**1F**) Pretreatment SS-OCT B-scan with choroidal thickness segmentation and (**2F**) corresponding posttreatment image demonstrating choroidal thinning after therapy

**Fig. 2:**
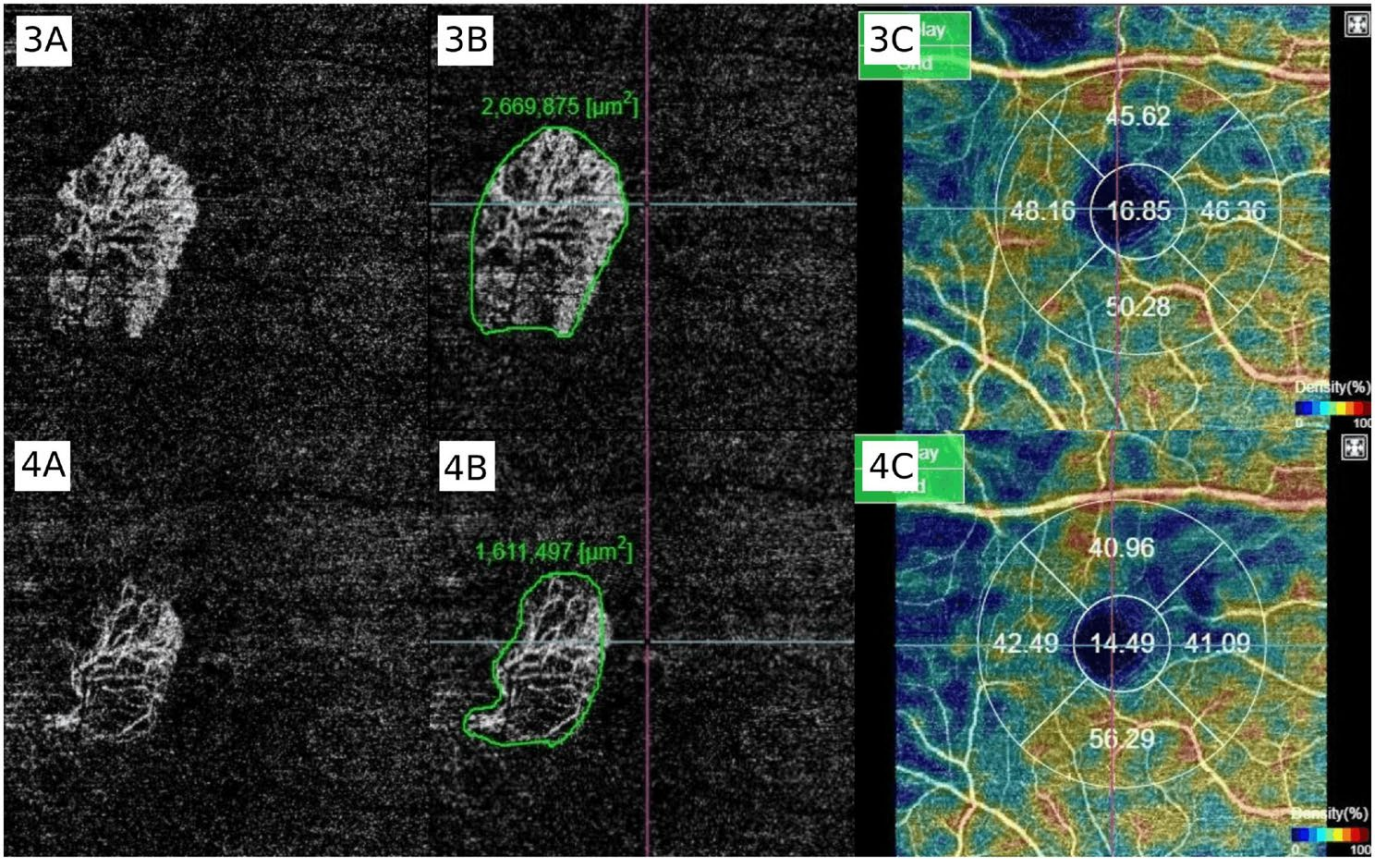
Images labeled with 3 correspond to the baseline evaluation, whereas images labeled with 4 correspond to the posttreatment evaluation (one month after the third aflibercept injection). (**3A**) SS-OCTA outer retina image demonstrating a macular neovascular membrane at baseline. (**4A**) Corresponding posttreatment SS-OCTA image showing a reduction in the vascular network complexity and overall lesion size. (**3B**) Quantitative delineation of the macular neovascularization area (MNVA) at baseline, measuring 2,668.87 mm², with a marked reduction in the posttreatment measurement (**4B**) to 1,611.48 mm². (**3C**) Pretreatment vessel density (VD) map demonstrating a central VD value of 16.85, with a further reduction observed in the posttreatment map (**4C**) (VD = 14.49)

**Fig. 3:**
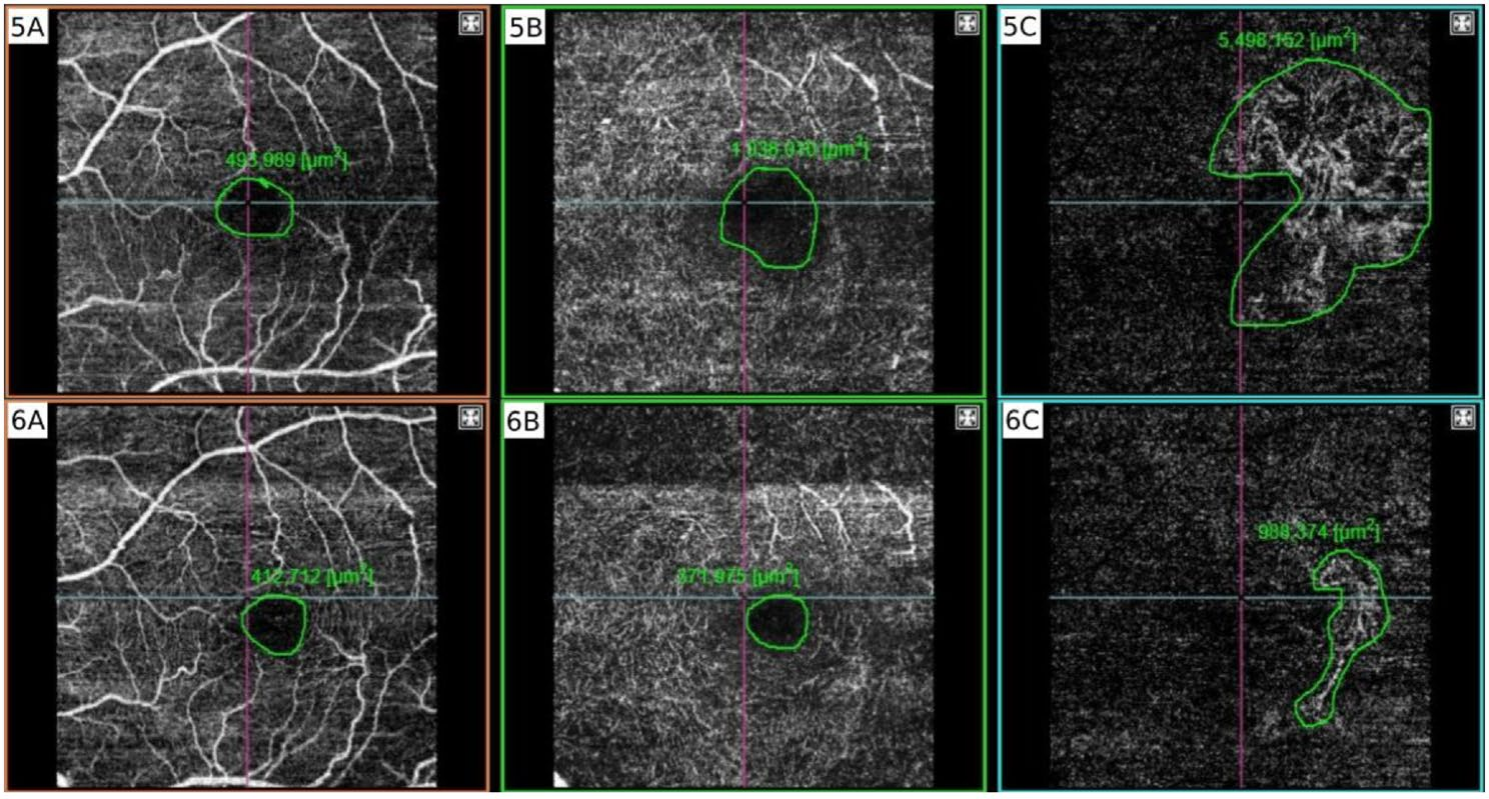
SS-OCTA of another patient of this study: images labeled with 5 correspond to the baseline evaluation, whereas images labeled with 6 correspond to the posttreatment evaluation (one month after the third aflibercept injection). (**5A**) SS-OCTA image of the superficial retinal plexus demonstrating the avascular area of the superficial plexus (AASP) at baseline, measuring 493.98 mm², with a reduction observed in the posttreatment image (**6A**) (412.71 mm²). (**5B**) Baseline avascular area of the deep plexus (AADP), measuring 1,038.070 mm², with a subsequent decrease in the posttreatment image (**6B**) (871.97 mm²). (**5C**) Another pretreatment delineation of the macular neovascularization area (MNVA), measuring 5,498.15 mm², with a marked reduction in lesion size in the posttreatment image (**6C**) (988.37 mm²), indicating a substantial decrease in neovascular area following therapy

Figures 1, 2 and 3 illustrate representative examples of biomarker measurements obtained before and after treatment with a standardized loading dose of aflibercept. The results of the SS-OCT-based assessment of structural biomarkers, including the CMT and CCT, in a treatment-naïve patient with nAMD are shown in Fig. 1. The evaluation of vascular biomarkers using SS-OCTA, specifically the MNVA and VD, in another patient from the same cohort is shown in Fig. 2. SS-OCTA-derived microvascular changes, highlighting reductions in the AASP and AADP, as well as an additional example of MNVA reduction following treatment, are shown in Fig. 3.

### Correlation analysis

In this study, several statistically significant correlations were identified among the pre- (baseline) and posttreatment (Post) biomarkers assessed by SS-OCT and SS-OCTA. Overall, the correlation strengths were distributed as follows: one very high correlation, one high correlation, eight moderate correlations, and four low correlations. The detailed characteristics of each correlation are (Table 4):

**Table 4:** Correlation coefficients (r), 95% confidence intervals, and p-values between structural, vascular, and functional biomarkers derived from SS-OCT and SS-OCTA in treatment-naïve eyes with neovascular AMD treated with aflibercept

#	Biomarker 1	Biomarker 2	*r*	95% CI (lower)	95% CI (upper)	*p*-value	Interpretation
1	*CMT pre (µm)*	*CMT post (µm)*	0.530	0.13	0.78	0.013	Moderate positive
2	*CMT pre (µm)*	*CMT reduction (µm)*	-0.951	-0.98	-0.88	< 0.001	Very high negative
3	*CCT pre (µm)*	*CCT post (µm)*	0.873	0.71	0.95	0.002	High positive
4	*BCVA pre (logMAR)*	*CMT pre (µm)*	0.589	0.21	0.81	0.005	Moderate positive
5	*BCVA pre (logMAR)*	*CMT reduction (µm)*	-0.497	-0.76	-0.08	0.022	Low negative
6	*BCVA post (logMAR)*	*CMT pre (µm)*	0.476	0.06	0.75	0.029	Low positive
7	*MNVA pre (mm²)*	*CCT post (µm)*	-0.506	-0.77	-0.10	0.019	Moderate negative
8	*AADP pre (mm²)*	*CMT pre (µm)*	0.592	0.22	0.82	0.005	Moderate positive
9	*AADP pre (mm²)*	*CMT reduction (µm)*	-0.581	-0.81	-0.20	0.006	Moderate negative
10	*AADP reduction (%)*	*CMT pre (µm)*	0.674	0.34	0.86	0.001	Moderate positive
11	*AADP reduction (%)*	*CMT reduction (%)*	0.558	0.17	0.80	0.009	Moderate positive
12	*AADP reduction (%)*	*CCT reduction (%)*	0.447	0.02	0.74	0.042	Low positive
13	*AASP reduction (%)*	*CCT reduction (µm)*	-0.582	-0.81	-0.20	0.006	Moderate negative
14	*AASP reduction (%)*	*CCT reduction (%)*	0.440	0.01	0.73	0.046	Low positive

BCVA: best-corrected visual acuity; logMAR: logarithm of the minimum angle of resolution; CMT: central macular thickness; CCT: central choroidal thickness; AASP: avascular area of the superficial plexus; AADP: avascular area of the deep plexus. MNVA: macular neovascularization area; CI: confidence interval. r = correlation coefficient

**Graph 1:**
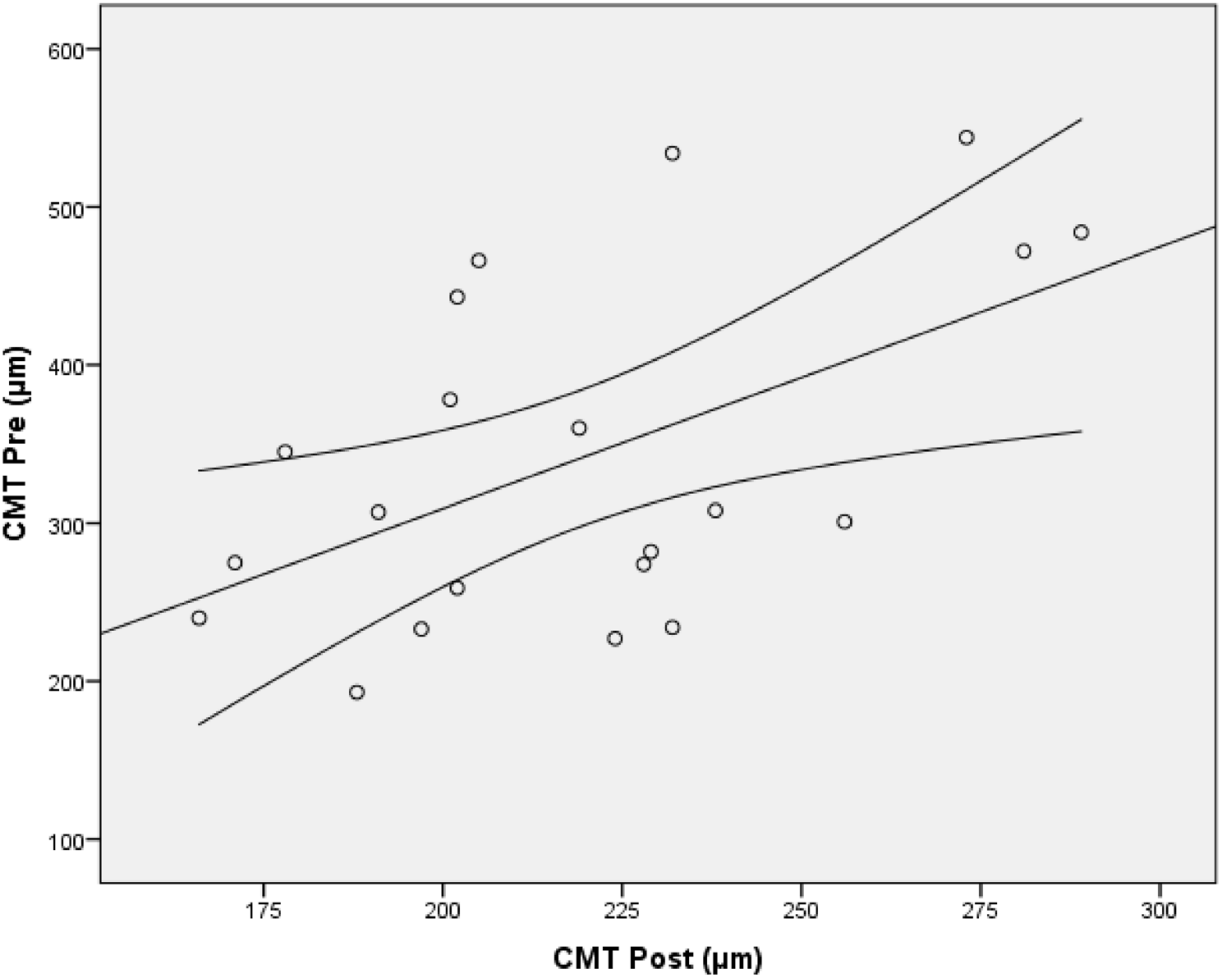
CMT Pre (µm): central macular thickness pretreatment, CMT Post (µm): central macular thickness posttreatment

**Graph 2:**
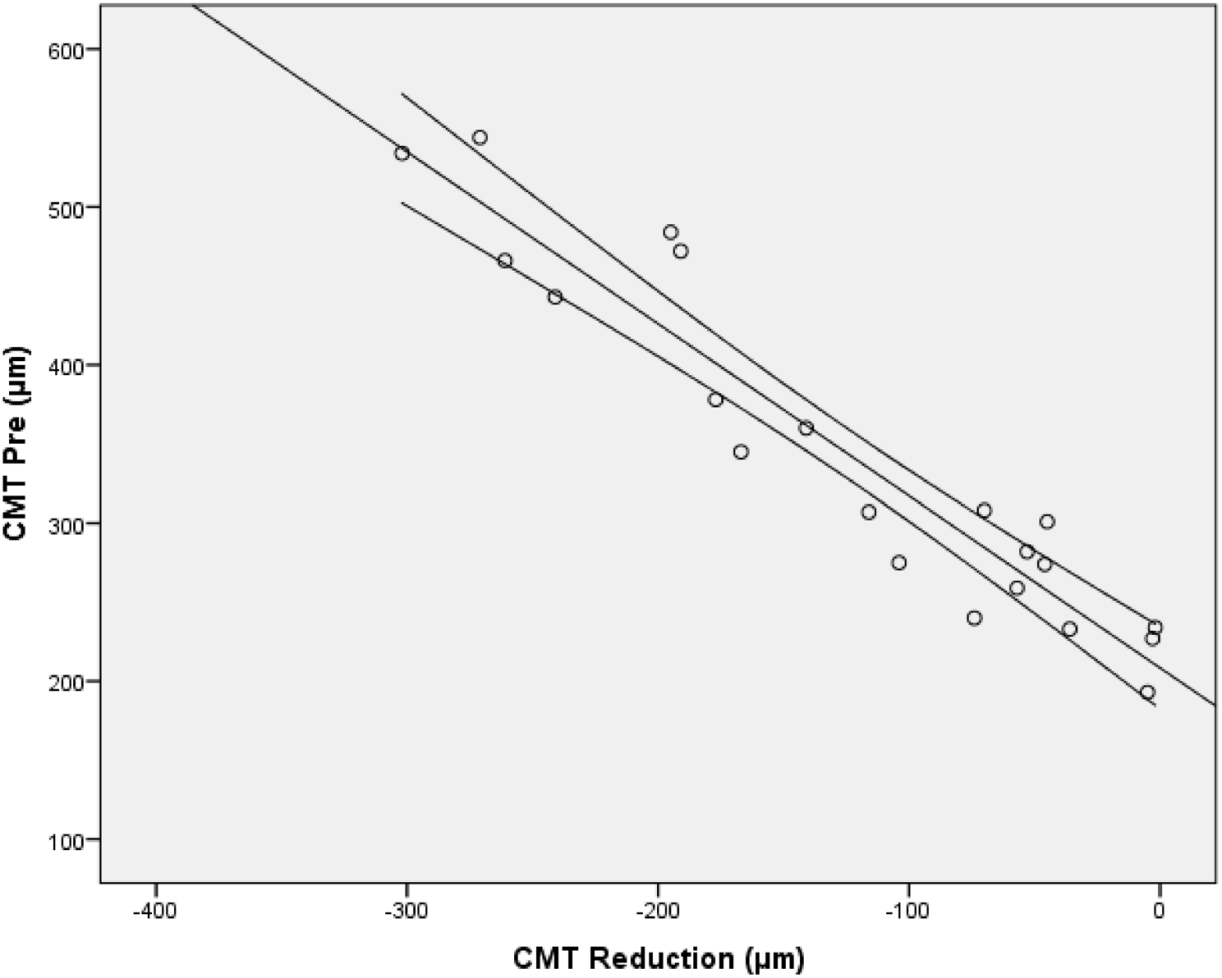
CMT Pre (µm): central macular thickness pretreatment, CMT Reduction (µm): reduction of central macular thickness

**Graph 3:**
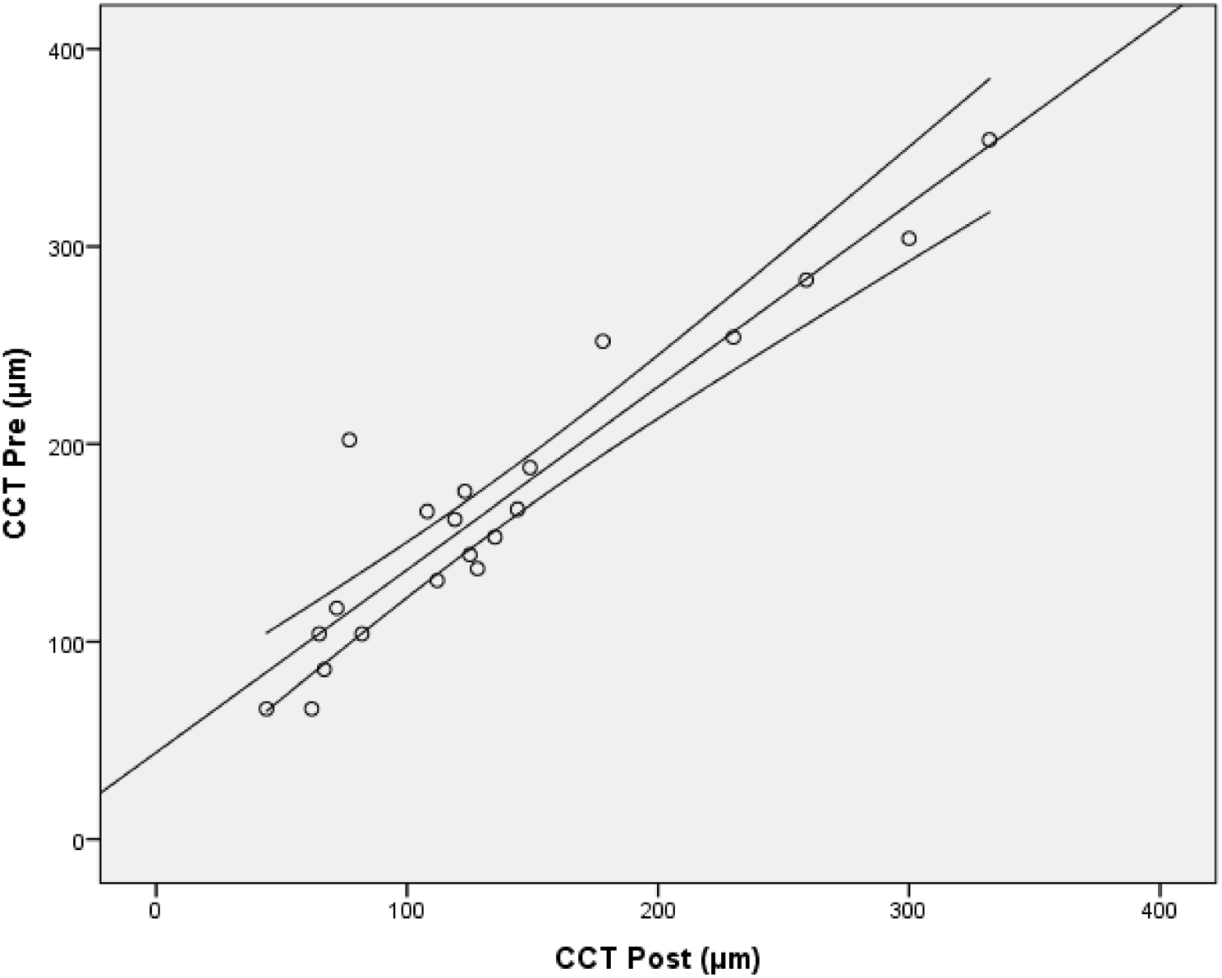
CCT Pre (µm): central choroidal thickness pretreatment, CCT Post (µm): central choroidal posttreatment

**Graph 4:**
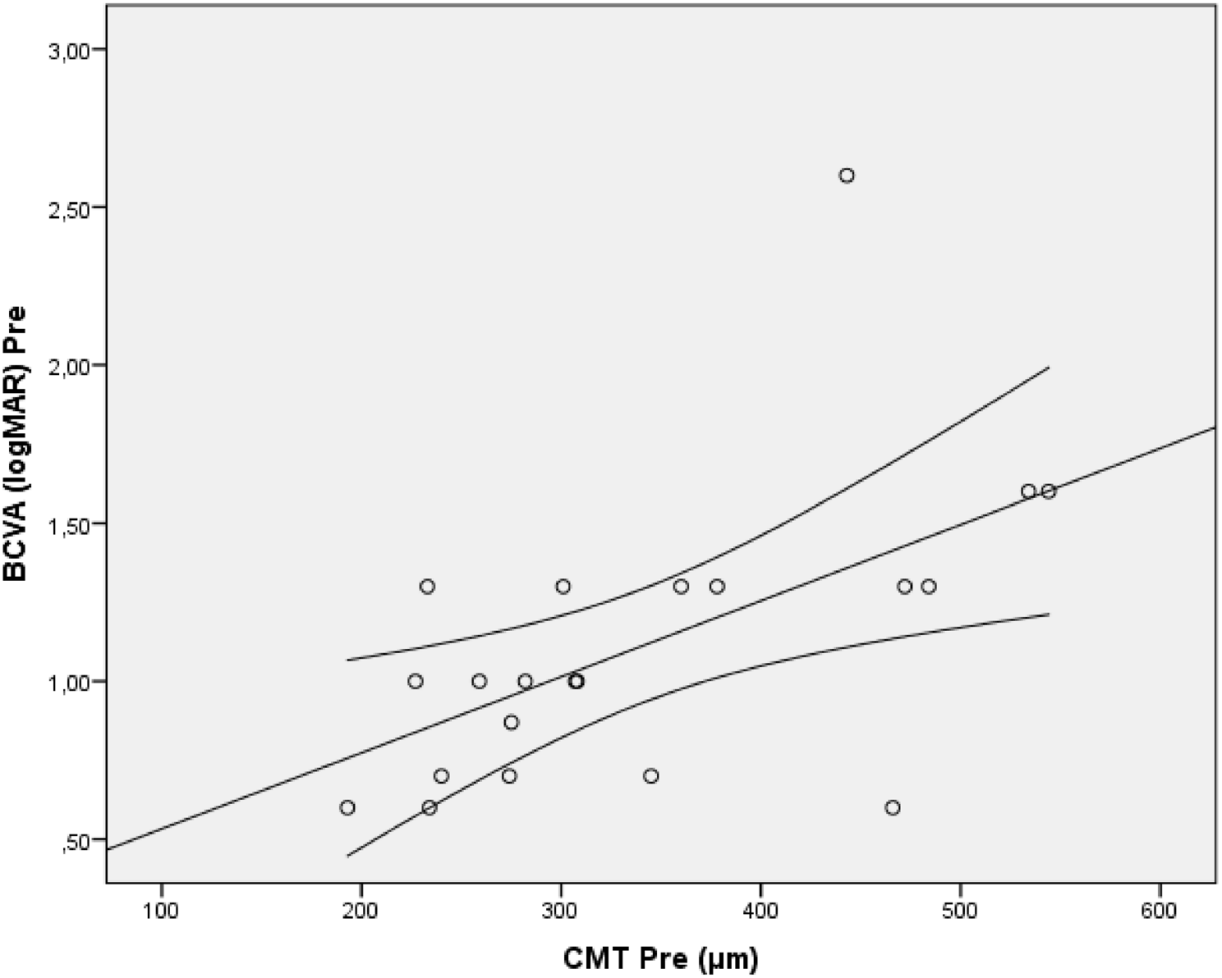
BCVA (logMAR) Pre: best-corrected visual acuity pretreatment, CCT Pre (µm): central choroidal thickness pretreatment

**Graph 5:**
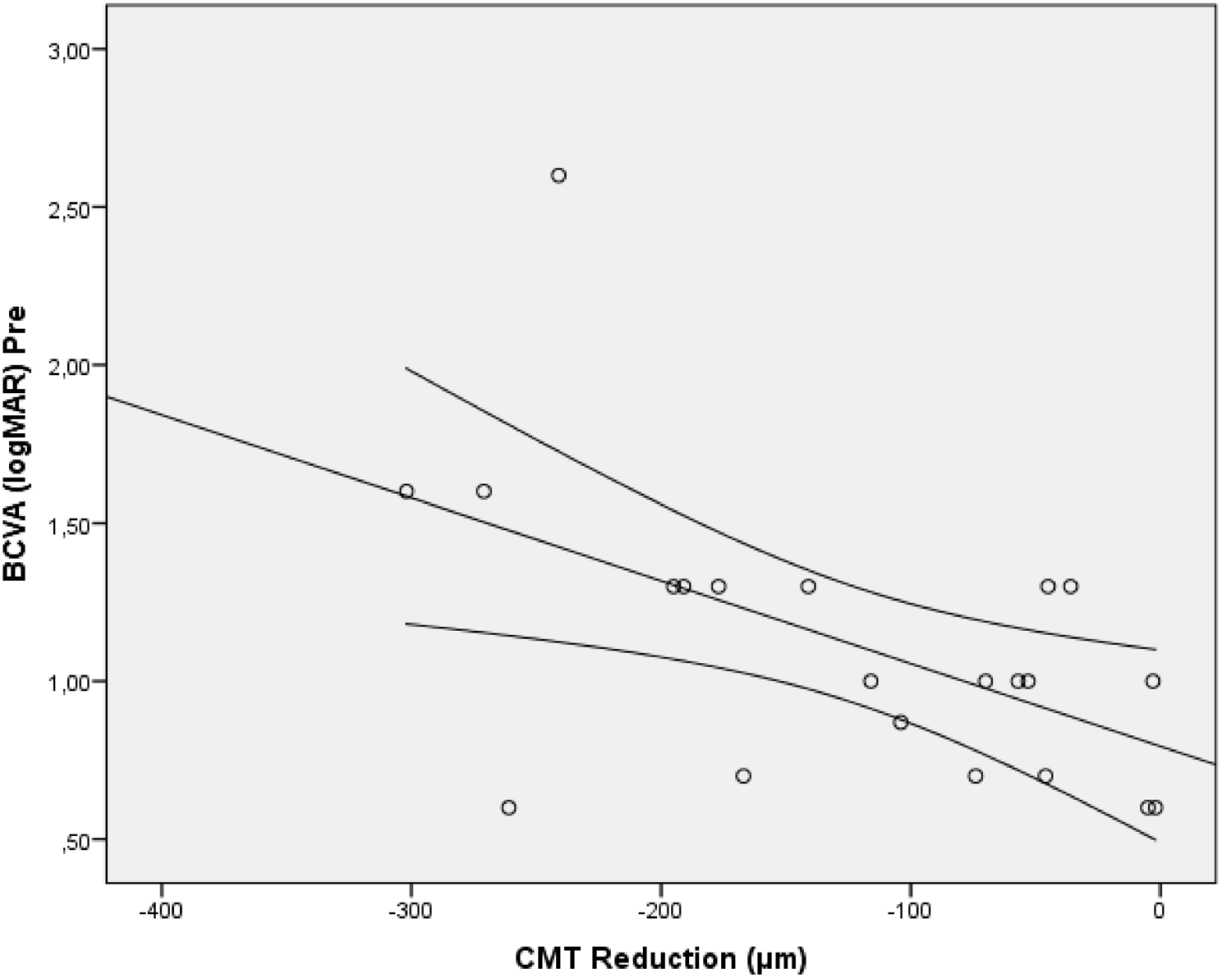
BCVA (logMAR) Pre: best-corrected visual acuity pretreatment, CMT Reduction (µm): reduction of central macular thickness

**Graph 6:**
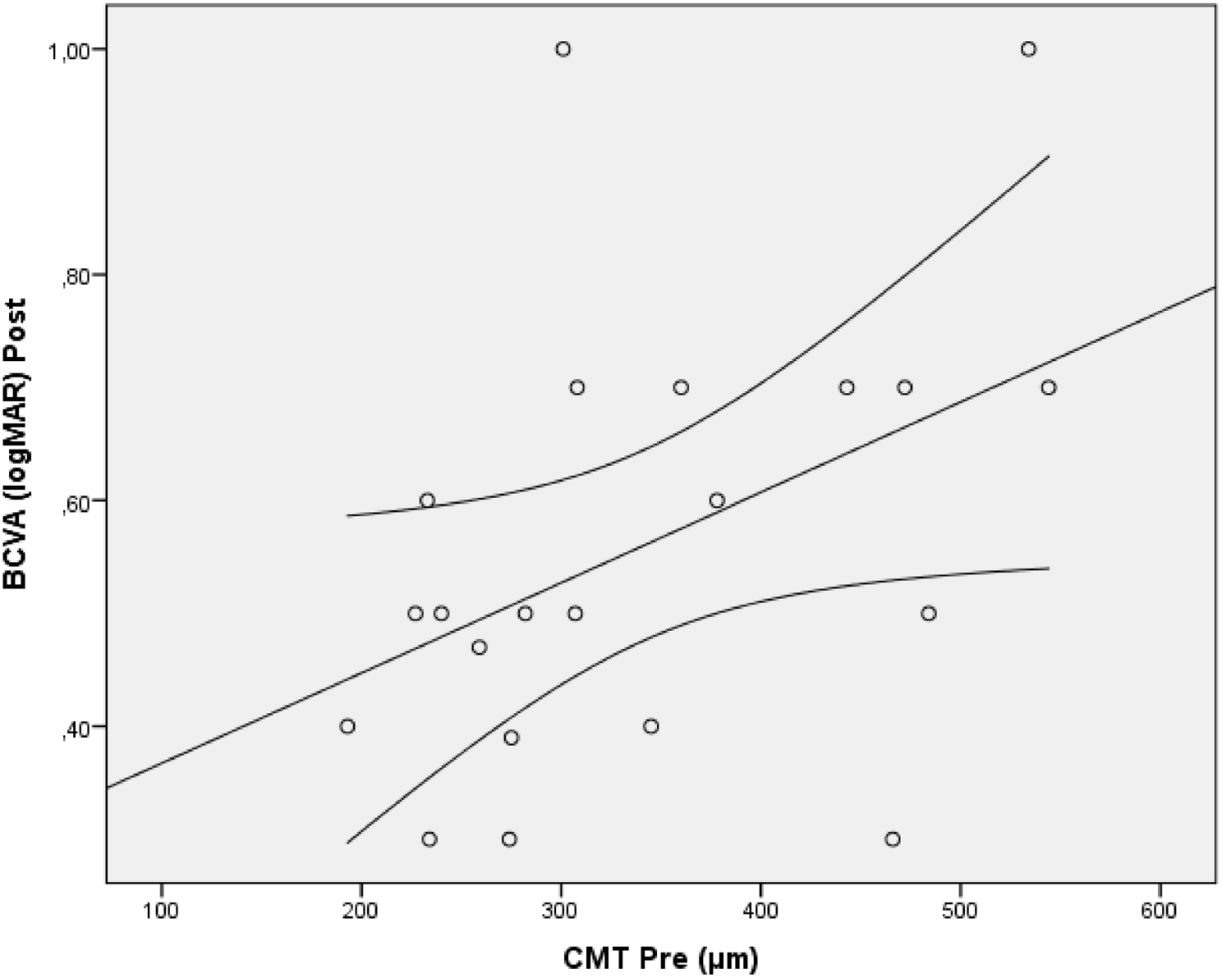
BCVA (logMAR) Post: best-corrected visual acuity posttreatment, CMT Pre (µm): central macular thickness pretreatment

**Graph 7:**
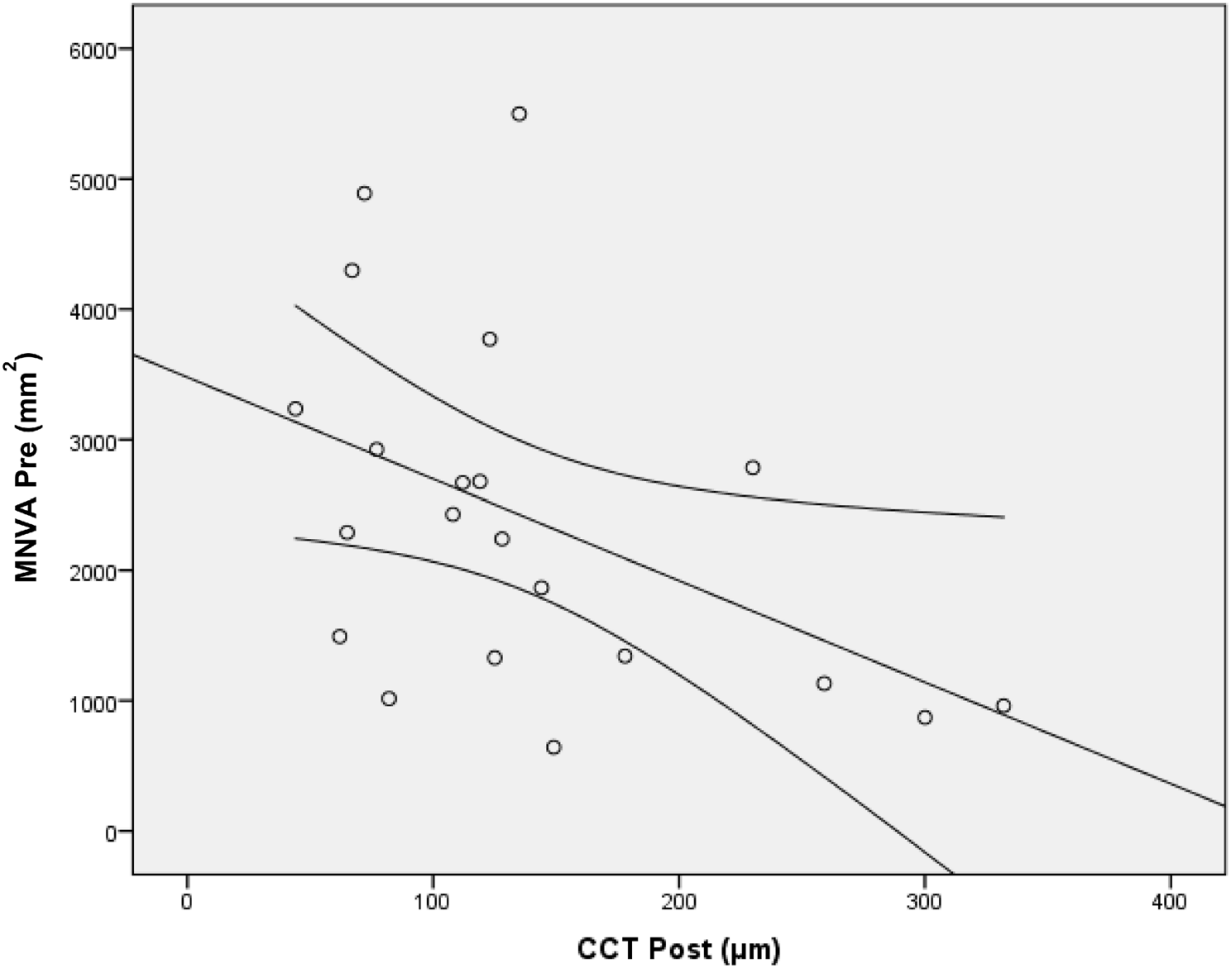
MNVA Pre (mm^2^): macular neovascularization area pretreatment, CCT Post (µm): central choroidal thickness posttreatment

**Graph 8:**
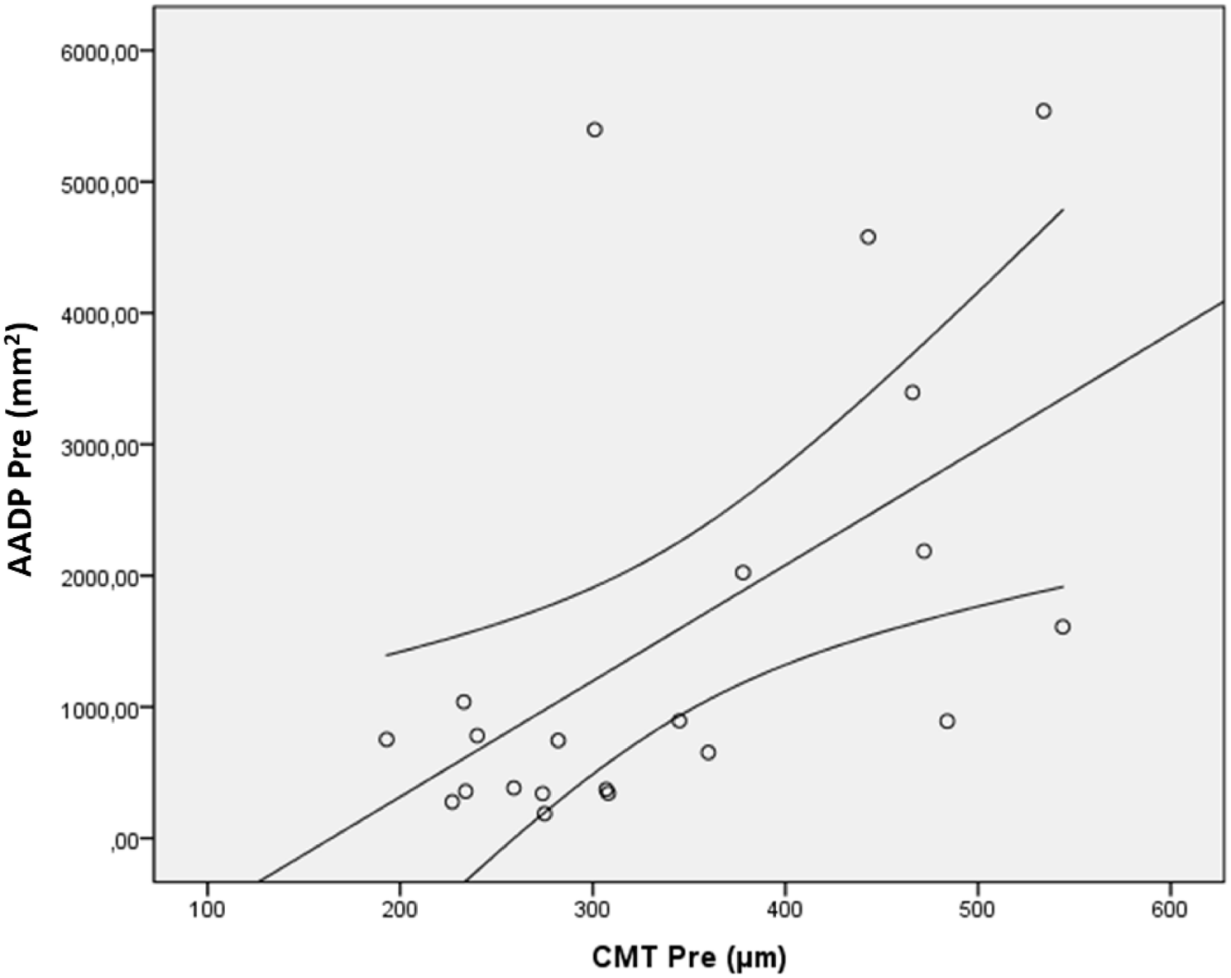
AADP Pre (mm^2^): avascular area of the deep plexus pretreatment, CMT Pre (µm): central macular thickness pretreatment

**Graph 9:**
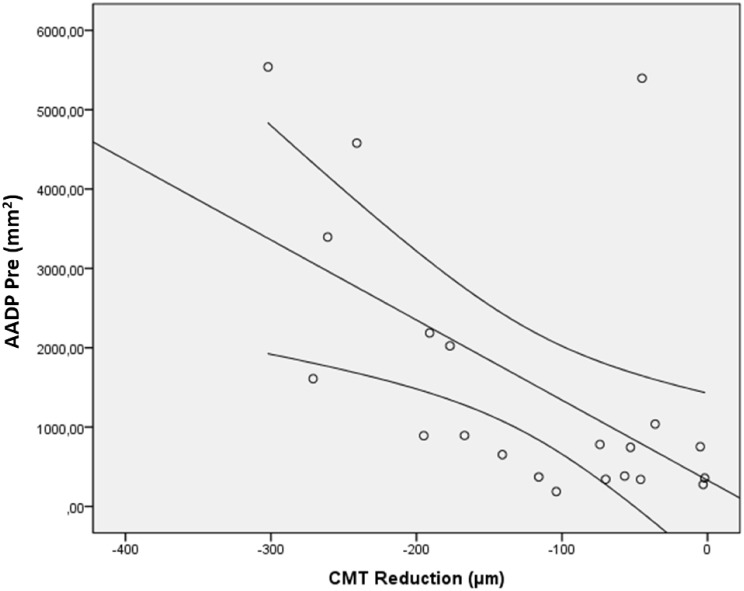
AADP Pre (mm^2^): avascular area of the deep plexus pretreatment, CMT Reduction (µm): reduction of central macular thickness

**Graph 10:**
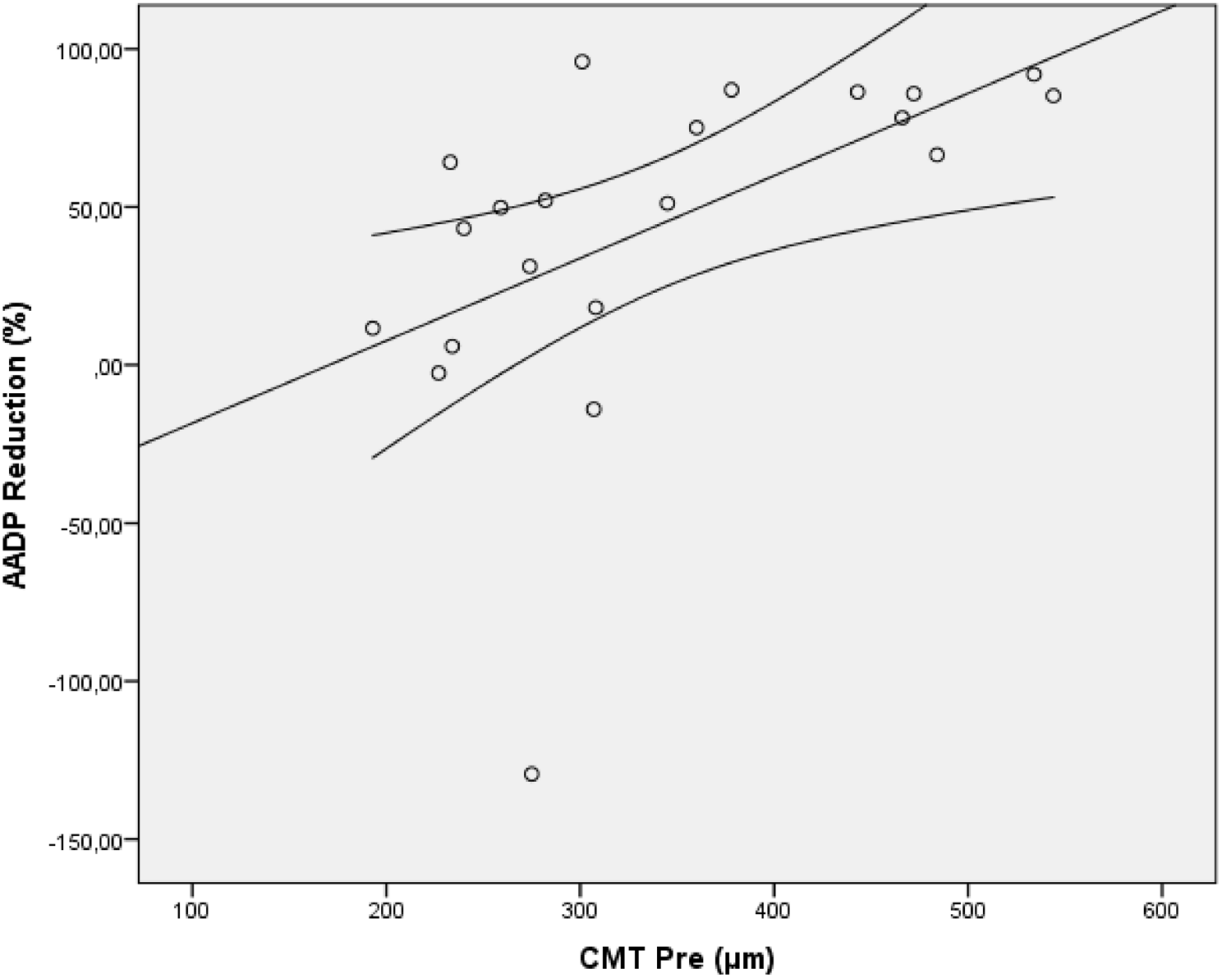
AADP Reduction (%): reduction of the avascular area of the deep plexus, CMT Pre (µm): central macular thickness pretreatment

**Graph 11:**
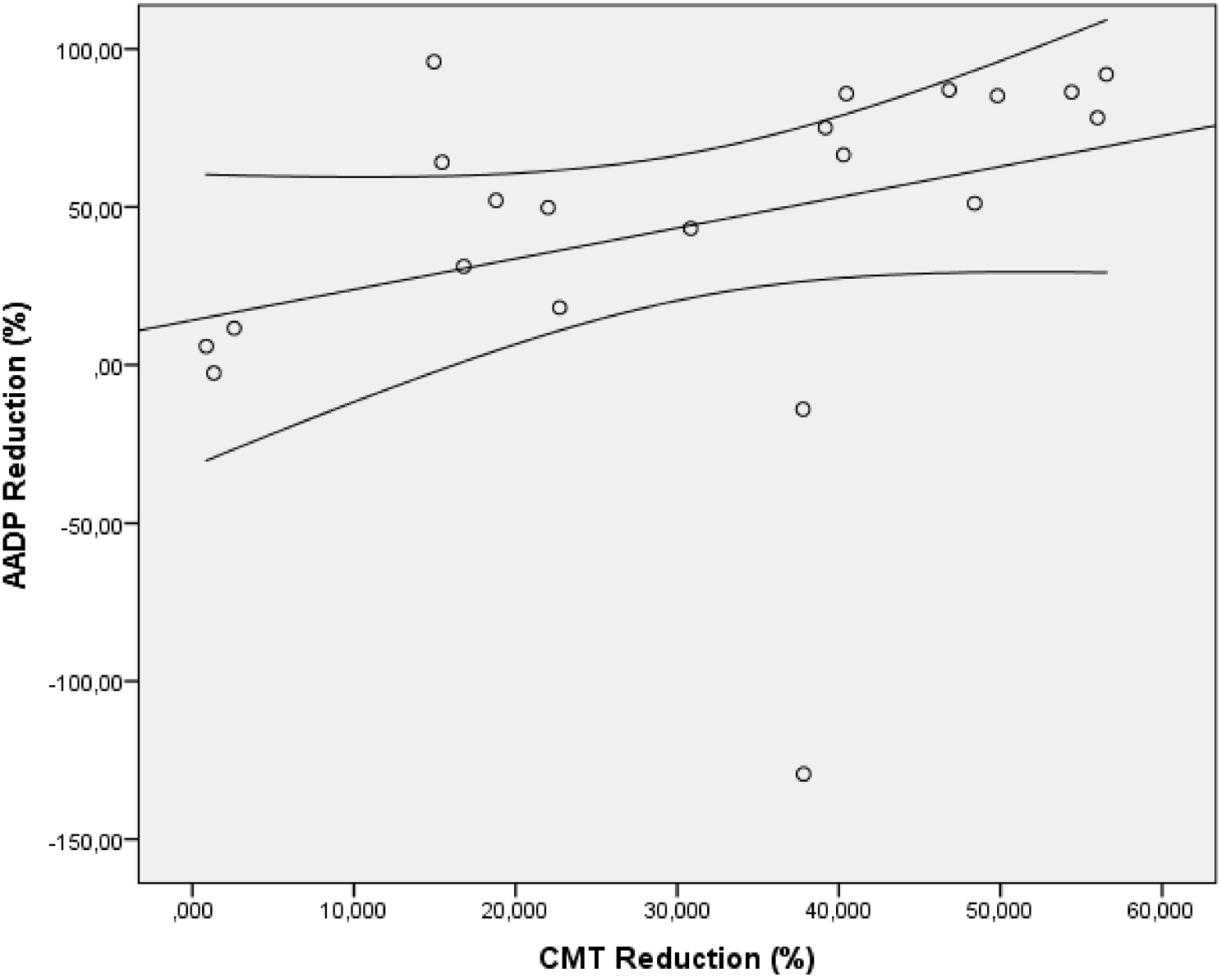
AADP Reduction (%): reduction of the avascular area of the deep plexus, CMT Reduction (%): reduction of the central macular thickness

**Graph 12:**
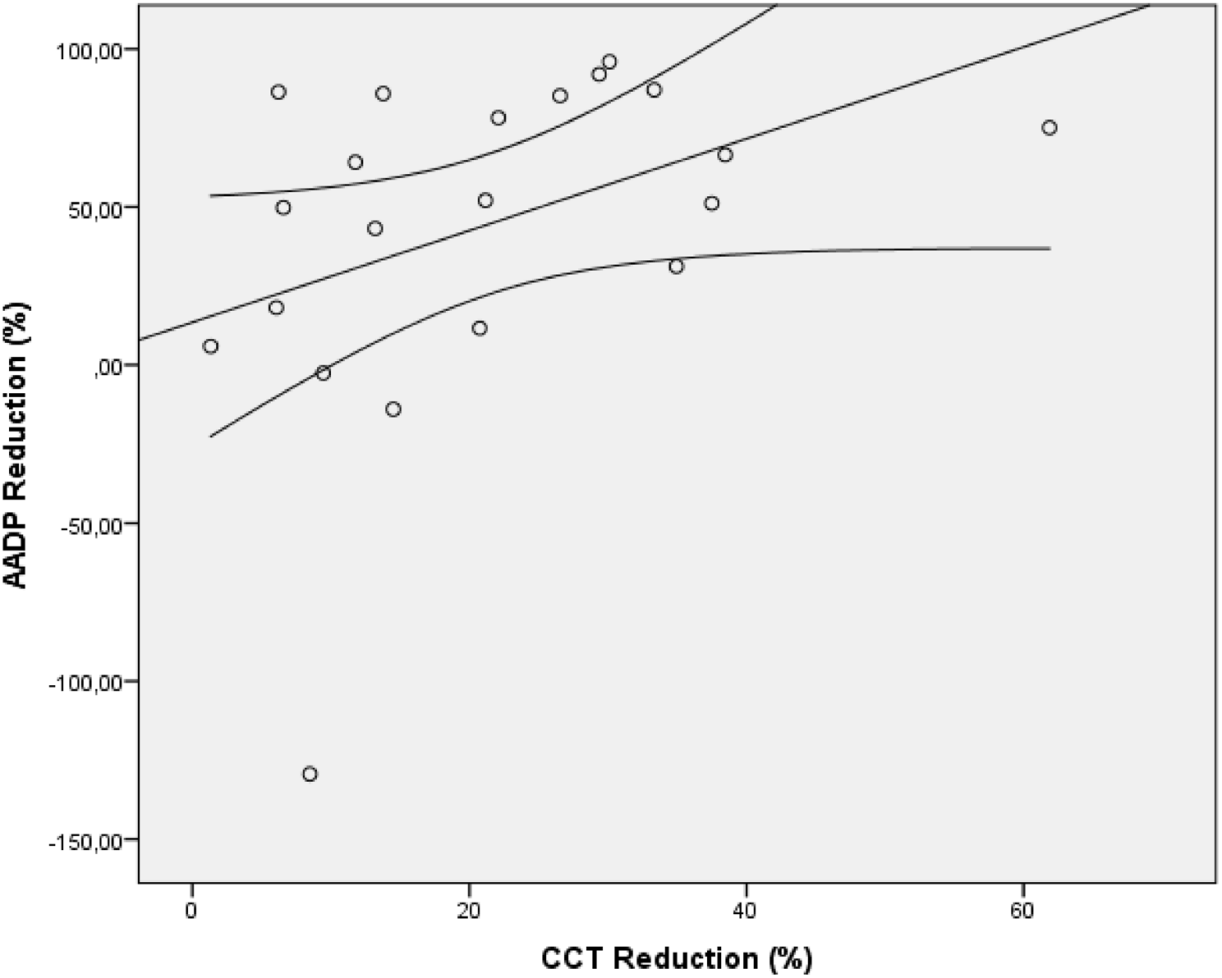
AADP Reduction (%): reduction of the avascular area of the deep plexus, CCT Reduction (%): reduction of the central choroidal thickness

**Graph 13:**
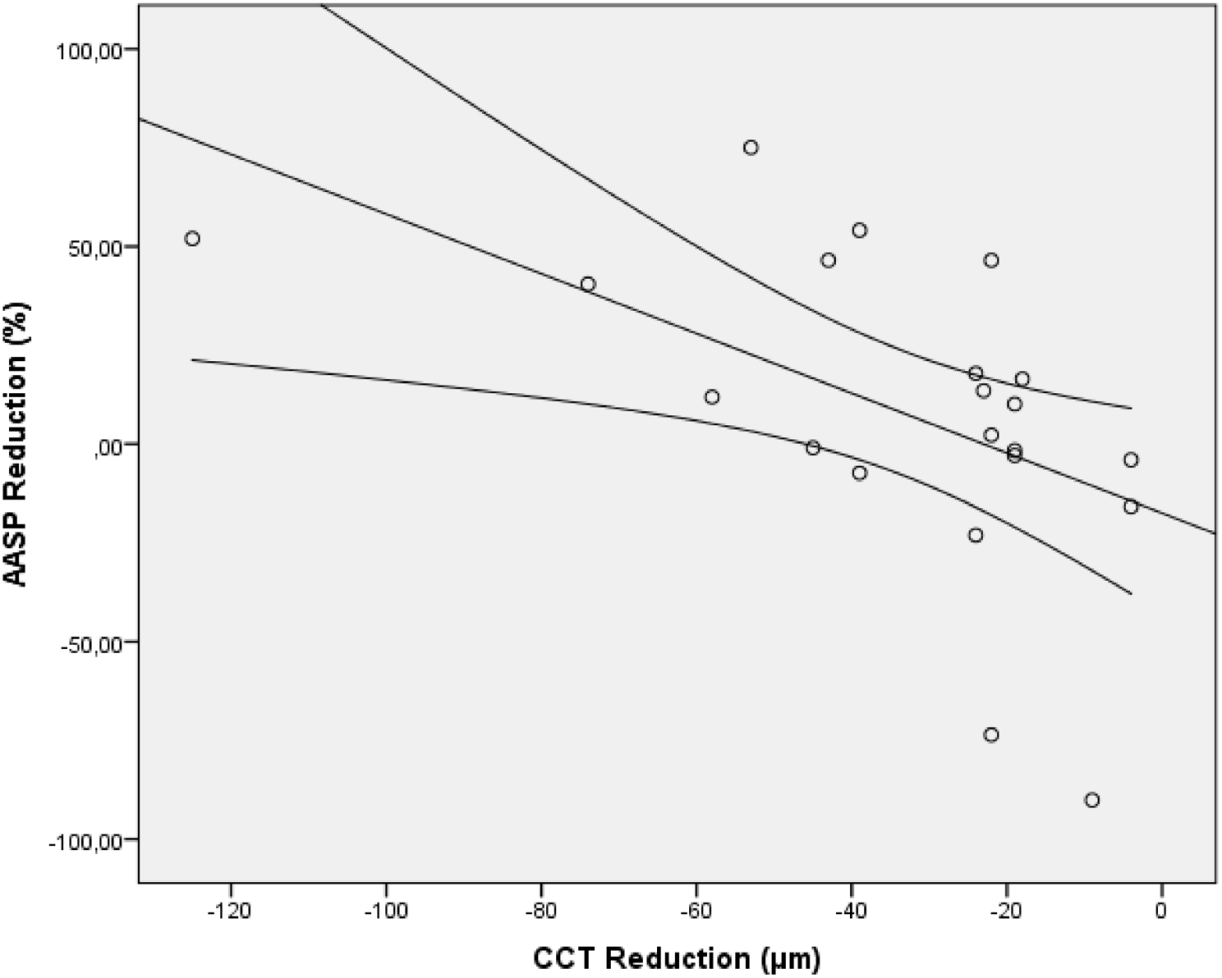
AASP Reduction (%): reduction of the avascular area of the superficial plexus, CCT Reduction (µm): reduction of the central choroidal thickness

**Graph 14:**
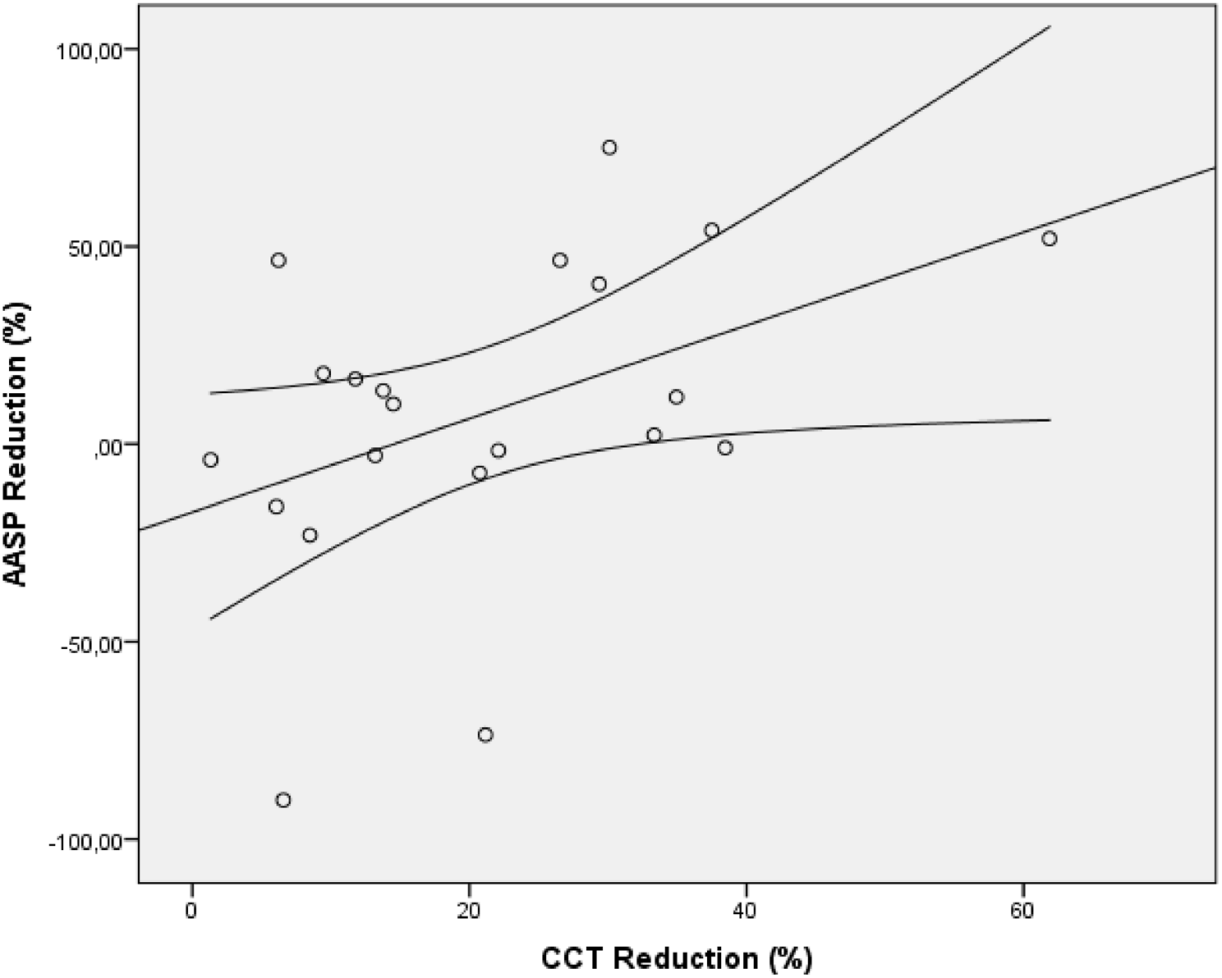
AASP Reduction (%): reduction of the avascular area of the superficial plexus, CCT Reduction (%): reduction of the central choroidal thickness

1. The *CMT Pre (µm) and the CMT Post (µm)* are significantly correlated (*p* = 0.013), and the correlation is 0.530 (graph 1), indicating a moderate positive correlation.
2. The *CMT Pre (µm) and the CMT Reduction (µm)* are significantly correlated (*p* < 0.000), and the correlation is -0.951 (graph 2), indicating a very high negative correlation.
3. The *CCT Pre (µm) and the CCT Post (µm)* are significantly correlated (*p* = 0.002), and the correlation is 0.873 (graph 3), indicating a high positive correlation.
4. The *BCVA (logMAR) Pre and the CMT Pre (µm)* are significantly correlated (*p* = 0.005), and the correlation is 0.589 (graph 4), indicating a moderate positive correlation.
5. The *BCVA (logMAR) Pre and the CMT Reduction (µm)* are significantly correlated (*p* = 0.022), and the correlation is -0.497 (graph 5), indicating a low negative correlation.
6. The *BCVA (logMAR) Post and CMT Pre (µm)* are significantly correlated (*p* = 0.029), and the correlation is 0.476 (graph 6), indicating a low positive correlation.
7. The *MNVA Pre (mm*^*2*^*) and the CCT Post (µm)* are significantly correlated (*p* = 0.019), and the correlation is -0.506 (graph 7), indicating a moderate negative correlation.
8. The *AADP (mm*^*2*^*) Pre and the CMT Pre (µm)* are significantly correlated (*p* = 0.005), and the correlation is 0.592 (graph 8), indicating a moderate positive correlation.
9. The *AADP (mm*^*2*^*) Pre and the CMT Reduction (µm)* are significantly correlated (*p* = 0.006), and the correlation is -0.581 (graph 9), indicating a moderate negative correlation.
10. The *AADP Reduction (%) and the CMT Pre (µm)* are significantly correlated (*p* = 0.001), and the correlation is 0.674 (graph 10), indicating a moderate positive correlation.
11. The *AADP Reduction (%) and the CMT Reduction (%)* are significantly correlated (*p* = 0.009), and the correlation is 0.558 (graph 11), indicating a moderate positive correlation.
12. The *AADP Reduction (%) and the CCT Reduction (%)* are significantly correlated (*p* = 0.042), and the correlation is 0.447 (graph 12), indicating a low positive correlation.
13. The *AASP Reduction (%) and the CCT Reduction (µm)* are significantly correlated (*p* = 0.006), and the correlation is -0.582 (see graph 13), indicating a moderate negative correlation.
14. The *AASP Reduction (%) and the CCT Reduction (%)* are significantly correlated (*p* = 0.046), and the correlation is 0.440 (graph 14), indicating a low positive correlation.

All the correlations evaluated in this study are listed in Table 5.

**Table 5:** All correlations between the different variables/biomarkers of SS-OCT and SS-OCTA

CMT Pre (µm)	CMT Pre (µm)	CMT Post (µm)	CMT Reduction (µm)	CCT Pre (µm)	CCT Post (µm)	CCT Reduction (µm)	BCVA Pre	BCVA Post	BCVA Reduction	MNVA Pre (mm2)	MNVA Post (mm2)	MNVA Reduction (mm2)	VD Pre	VD Post	VD Reduction	AADP Pre (mm2)	AADP Post (mm2)	AADP Reduction (mm2)	AASP Pre (mm2)	AASP Post (mm2)	AASP Reduction (mm2)
CMT Post (µm)	0.53																				
	**0.0130**																				
CMT Reduction (µm)	0.83	0.96																			
	**0.000003**	**0.0130**																			
CCT Pre (µm)	-0.42	-0.06	0.03																		
	0.8560	0.7820	0.9150																		
CCT Post (µm)	-0.35	-0.09	0.26	0.87																	
	0.1220	0.7010	0.2580	**0.002**																	
CCT Reduction (µm)	0.34	0.20	0.28	-0.28	-0.59																
	0.1310	0.386	0.2280	0.215	**0.005**																
BCVA Pre	0.59	0.42	-0.50	0.15	0.67	-0,32															
	**0.0050**	0.0550	**0.0220**	0.5270	0.7730	0.1600															
BCVA Post	0.48	0.42	-0.35	0.09	0,00	-0,26	0,86														
	**0**,**0290**	0,0580	0,1190	0,7050	0.9950	0.2550	**0.0050**														
BCVA Reduction	0.28	0.07	-0.30	0.28	0.10	0.00	0.26	-0.24													
	0.2130	0.7780	0.1860	0.2210	0.6810	0.9890	0.2590	0.3030													
MNVA Pre (mm2)	0.19	0.25	-0.13	-0.42	-0.51	-0.19	0.29	0.18	0.17												
	0.4050	0.2850	0.5750	0.0600	**0.0190**	0.4050	0.1970	0.4350	0.4760												
MNVA Post (mm2)	-0.08	-0.08	0.12	-0.31	-0.48	-0.09	-0.18	-0.26	0.09	0.73											
	0.7240	0.7200	0.6140	0.1780	**0.0290**	0.6860	0.4320	0.2500	0.6910	**0.0002**											
MNVA Reduction (mm2)	0.32	0.33	-0.24	-0.01	0.12	-0.13	0.62	0.58	0.02	0.11	-0.56										
	0.1590	0.1440	0.2860	0.9820	0.6140	0.5710	**0.0030**	**0.0060**	0.9170	0.6330	**0.0080**										
VD Pre	0.32	0.02	-0.27	-0.24	-0.29	-0.03	0.05	0.14	-0.20	0.27	0.21	-0.11									
	0.1630	0.9200	0.2360	0.2910	0.2030	0.8880	0.8220	0.5530	0.3750	0.2340	0.3720	0.6300									
VD Post	0.04	0.22	0.06	0.25	0.09	-0.09	0.09	-0.07	0.35	0.50	0.41	-0.09	0.29								
	0.8800	0.3320	0.8140	0.2700	0.6990	0.6900	0.6870	0.7630	0.1160	**0.0220**	0.0670	0.6950	0.1990								
VD Reduction	0.27	0.01	-0.31	-0.31	-0.27	0.12	0.03	0.22	-0.34	-0.16	-0.13	0.08	0.75	-0.35							
	0.2340	0.9530	0.1780	0.1780	0.2290	0.6170	0.8980	0.3480	0.1330	0.5030	0.5900	0.7470	**0.0001**	0.1160							
AADP Pre (mm2)	0.59	0.21	-0.58	-0.08	-0.06	-0.21	0.53	0.54	-0.12	0.21	-0.17	0.45	0.36	-0.07	0.43						
	**0.0050**	0.3570	**0.0060**	0.7450	0.8140	0.3570	**0.0140**	**0.0120**	0.6090	0.3130	0.4710	**0.0420**	0.1130	0.7630	0.0530						
AADP Post (mm2)	0.07	-0.46	-0.25	0.16	0.28	0.21	-0.31	-0.27	-0.12	-0.20	-0.30	-0.20	-0.02	-0.36	0.17	0.25					
	0.7650	**0.0370**	0.2800	0.4930	0.2140	0.3600	0.1730	0.2320	0.6150	0.3810	0.1880	0.3960	0.9420	0.1150	0.4500	0.2760					
AADP Reduction (mm2)	0.67	0.41	-0.58	-0.08	-0.18	-0.35	0.69	0.66	-0.04	0.35	-0.05	0.29	0.38	0.10	0.25	0.90	-0.12				
	**0.0010**	0.0630	**0.0060**	0.7240	0.4240	0.1210	**0.0010**	**0.0010**	0.8710	0.1190	0.8230	0.2040	0.0900	0.6830	0.2800	**0.0002**	0.5940				
AASP Pre (mm2)	0.18	-0.32	-0.34	0.14	0.11	-0.22	0.19	0.26	-0.15	0.06	-0.17	0.24	0.11	-0.08	0.15	0.63	0.55	0.41			
	0.4270	0.1590	0.1310	0.5350	0.6220	0.3370	0.4200	0.2500	0.5240	0.7840	0.4610	0.2910	0.6380	0.7290	0.5260	**0.0020**	**0.0100**	0.0670			
AASP Post (mm2)	-0.20	-0.49	-0.06	-0.11	0.22	0.42	-0.16	-0.20	0.12	-0.27	-0.26	-0.04	-0.25	-0.24	-0.04	0.16	0.64	-0.07	0.43		
	0.3820	**0.0250**	0.8140	0.6340	0.3390	**0.0600**	0.4910	0.3800	0.6150	0.2320	0.2480	0.8760	0.2780	0.2890	0.8710	0.5040	**0.0020**	0.7500	0.0540		
AASP Reduction (mm2)	0.38	0.18	-0.37	0.27	-0.10	-0.58	0.53	0.52	-0.12	0.24	0.14	0.23	0.28	0.20	0.07	0.50	0.01	0.53	0.51	-0.46	
	0.0920	0.4280	0.1040	0.2400	0.9640	**0.0060**	**0.0140**	**0.0150**	0.5940	0.2920	0.5330	0.3210	0.2180	0.3820	0.7760	**0.0220**	0.9810	**0.0130**	**0.0190**	**0.0350**	

BCVA: best-corrected visual acuity; logMAR: logarithm of the minimum angle of resolution; CMT: central macular thickness; CCT: central choroidal thickness; AASP: avascular area of the superficial plexus; AADP: avascular area of the deep plexus. MNVA: macular neovascularization area; CI: confidence interval. r = correlation coefficient

## Discussion

### Overall results

In the present study, substantial anatomical and functional improvements were observed following the loading phase, as demonstrated in Table 3. Significant reductions in the BCVA (logMAR), CMT, CCT, MNVA, VD, AASP and AADP were documented, confirming the expected therapeutic effect of aflibercept on both the retinal and choroidal compartments. The BCVA and CMT results are consistent with those of prior reports in the literature [9, 17–19], which similarly emphasize pre- and posttreatment differences as indicators of treatment efficacy in nAMD patients. Lim et al. [17] reported significant postloading improvements in retinal morphology and the overall BCVA in treatment-naïve nAMD patients, confirming the expected early therapeutic effect of aflibercept. While both studies demonstrate comparable anatomical reductions following the loading phase, an important distinction is that their analysis focused primarily on the postloading central subfield thickness (CST) as the key predictive biomarker of treatment demand, whereas our work incorporated a broader set of structural, choroidal, and SS-OCTA-derived parameters. This multimodal approach allowed us to capture additional aspects of disease behavior that were not addressed in the prior study, thereby enhancing prognostic interpretation.

### Correlations

In addition to evaluating the response before and after treatment, we assessed the correlations between biomarkers. To our knowledge, this is the first prospective study to systematically evaluate correlations between SS-OCT and SS-OCTA-derived structural and vascular biomarkers both at baseline and after a standardized aflibercept loading dose in treatment-naïve nAMD patients. These findings provide new insight into how retinal and choroidal compartments interact during the first four months of aflibercept therapy, addressing a knowledge gap in the current literature.

In the present Discussion, we focused primarily on the correlations that demonstrated the greatest clinical and biological relevance to maintain clarity, avoid excessive length, and adhere to the reporting standards and structural guidelines of this scientific journal. And because multiple correlations were explored, the potential for type I error cannot be excluded. However, given the exploratory nature of this prospective study, the analyses were intended to identify biologically plausible associations between structural and vascular biomarkers rather than to establish definitive causal relationships.

### Structural retinal biomarkers: CMT

A moderate correlation was observed between CMT Pre and CMT Post (*r* = 0.530; *p* = 0.013), indicating that eyes with greater exudative thickening at pretreatment tended to maintain relatively greater retinal thickness even after the loading phase (item 1). The CMT Pre demonstrated a very high negative correlation with the magnitude of CMT reduction following treatment (*r* = − 0.951; *p* < 0.001), revealing that eyes with the thickest retinas at baseline experienced the greatest absolute anatomical reduction after treatment (item 2). This result underscores the pivotal role of the CMT as a structural biomarker in nAMD, both in terms of defining the initial disease burden and serving as a candidate biomarker associated with the magnitude of the therapeutic response. Clinically, these findings suggest that the CMT Pre may help anticipate treatment trajectory: eyes presenting with substantial exudation are more likely to exhibit pronounced anatomical recovery because of a reduction in thickness, whereas eyes with lower baseline thickness values may show more limited structural changes, supporting individualized expectations and monitoring strategies.

These results are supported by the PRECISE Study Report 3 [20], which reported that increased CST Pre is a strong determinant of persistent anatomical activity following an aflibercept loading dose in treatment-naïve nAMD patients. In that large real-world cohort, a higher CST Pre was independently associated with the presence of early residual fluid after the loading dose was administered, indicating that eyes with greater initial exudative burden tend to maintain greater retinal thickness or fluid activity despite treatment initiation. In this context, our observation that the CMT Pre is positively associated with the posttreatment thickness and that this value could predict a greater magnitude of anatomical reduction is consistent with the PRECISE report 3 findings, reinforcing that the CMT Pre could be a robust marker of disease burden and a key driver of early anatomical response to treatment. In contrast to our findings, namely, that the CMT Pre showed both a meaningful positive association with the posttreatment thickness and a very strong negative correlation with the magnitude of anatomical improvement, the post hoc analysis of the VIEW 1 and VIEW 2 trials [19] revealed only low or negligible correlations between CST changes and BCVA changes across all treatment arms and time points. Although the CST remains a widely used anatomical parameter in clinical practice, the results of the VIEW analysis demonstrated that CST fluctuations accounted for only 11% of the variance in the visual acuity (VA), indicating the limited ability of this biomarker in predicting functional outcomes. In light of these limitations, we deliberately expanded the analytical scope to include additional biomarkers, such as choroidal thickness parameters and SS-OCTA-derived neovascular and vascular features, aiming to capture complementary structural and perfusion information that may better reflect disease activity and therapeutic response. This broader approach underscores the importance of multimodal biomarker integration when treatment trajectories are evaluated in patients with nAMD.

Beyond retinal thickness parameters, choroidal structural characteristics also demonstrated important associations with treatment response.

### Choroidal structural biomarker: CCT

A highly significant positive correlation was identified between the CCT Pre and CCT Post (*r* = 0.873; *p* = 0.002), indicating that eyes with thicker choroids at baseline tended to maintain a proportionally greater choroidal thickness even after the loading phase (item 3). This high linear association suggests that choroidal structural characteristics in treatment-naïve nAMD eyes remain highly conserved despite VEGF suppression, indicating a degree of anatomical stability or an intrinsic choroidal phenotype that persists over time. The magnitude of this correlation is clinically meaningful: a coefficient of this strength indicates that the CCT Pre could serve as a candidate biomarker associated with posttreatment choroidal status, supporting its use as a candidate biomarker associated with posttreatment choroidal behavior. In clinical terms, these findings reinforce the relevance of assessing baseline choroidal thickness not only for disease characterization but also for anticipating long-term structural patterns following aflibercept therapy, potentially aiding in prognostic counseling and individualized treatment planning.

In agreement with our findings, the findings of both Boscia et al. [21] and Kumar et al. [22] provide complementary insights into choroidal behavior in treatment-naïve nAMD patients; however, these findings have important distinctions. Boscia et al. demonstrated that subfoveal choroidal thickness significantly decreases after aflibercept loading, confirming that VEGF suppression induces measurable choroidal contraction. Kumar et al. reported that baseline choroidal thickness is relatively stable over time but strongly associated with long-term treatment burden, with thicker choroids requiring substantially more injections at one year. While all three studies reveal that choroidal thickness is a relevant structural parameter in nAMD, importantly, our work demonstrates the *high linear correlation* of choroidal architecture before and after treatment (*r* = 0.873) after aflibercept loading, a relationship that has not been quantified in previous reports.

In addition to structural parameters, the relationship between retinal thickness and functional outcomes was also examined.

### Structure–function relationship: BCVA and CMT

Multiple significant associations were observed between the BCVA and CMT, reinforcing their close functional and structural interplay in nAMD. The BCVA Pre was moderately positively correlated with the CMT Pre (*r* = 0.589; *p* = 0.005), indicating that poorer VA at presentation was associated with greater exudative retinal thickening (item 4). However, the BCVA Pre showed a low negative correlation with the magnitude of CMT reduction after treatment (*r* = − 0.497; *p* = 0.022), suggesting that eyes with worse initial vision tended to exhibit more pronounced anatomical improvement during the loading phase (item 5). Additionally, the BCVA Post showed a low and positive correlation with the CMT Pre (*r* = 0.476; *p* = 0.029), indicating that a lower exudative burden at presentation was associated with a better VA after the loading phase (item 6). In other words, eyes with thinner retinas at baseline tended to achieve more favorable functional outcomes following therapy. These findings are consistent and reinforce the findings in item 4. Together, these correlations reinforce the strong functional and structural relationship between the CMT and BCVA, both at presentation and after treatment. The fact that these distinct correlations between the BCVA and CMT reached statistical significance underscores the strong interconnectedness of these two core biomarkers. Clinically, these findings reinforce the importance of integrating structural SS-OCT parameters with functional outcomes for prognostic assessment, suggesting that the CMT Pre provides meaningful insight into not only initial visual impairment but also the expected trajectory of functional recovery following aflibercept therapy.

Findings from the PRECISE Study Report 2 [23] further support the structural and functional associations identified in our cohort. This prior study demonstrated that increased CST was highly associated with poorer presentation and that the foveal IRF, subretinal hyperreflective material, atrophy, and fibrosis were independently correlated with worse baseline VA in treatment-naïve nAMD patients. In contrast, the presence of subretinal drusenoid deposits was associated with reduced odds of severe visual impairment, whereas the foveal SRF was not significantly associated with functional impairment after multivariable adjustment [22]. These results align with prior evidence demonstrating measurable structural and functional associations in patients with nAMD. Ou et al. [24] reported a statistically significant but small correlation between the baseline BCVA and central retinal thickness (CRT) in pooled age-related macular degeneration (AMD) trial data (*r* = − 0.24; *p* = 0.017), whereas longitudinal correlations between CRT changes and vision at 12 months were negligible, underscoring the limited predictive value of the CRT alone in this disease context. Complementing these findings, Nanji et al. [25] conducted the largest meta-analysis to date and demonstrated that several baseline OCT biomarkers, including the IRF, foveal intraretinal fluid, and subretinal hyperreflective material, were significantly associated with worse 12-month visual outcomes, whereas the baseline CST showed small but statistically significant associations with VA changes, including both positive and negative associations depending on the degree of thickening.

Taken together, these studies reinforce that OCT structural parameters exert a quantifiable influence on functional outcomes, corroborating the clinical relevance of the correlations between the BCVA and CMT identified in our cohort. Furthermore, these studies reinforce the heterogeneity of structure‒function correlations in different clinical contexts and support the rationale for incorporating complementary biomarkers derived from the choroid and SS-OCTA, as performed in our investigation, to improve the prognostic assessment of treatment-naïve nAMD patients.

We next explored whether neovascular complexity measured by OCTA was associated with choroidal structural remodeling.

### Neovascular burden and choroidal response: MNVA and CCT

A moderate negative correlation was identified between the MNVA Pre and CCT Post (*r* = − 0.506; *p* = 0.019), indicating that eyes presenting with larger neovascular membrane complexes at baseline tended to exhibit greater choroidal thinning following the loading phase (item 7). These findings suggest that the magnitude of neovascular involvement may influence the degree of choroidal structural remodeling after treatment. Clinically, this relationship is particularly relevant because it implies that the initial neovascular burden may be associated with more pronounced choroidal thinning after aflibercept treatment.

This specific quantitative correlation between MNVA Pre and CCT Post appears to represent a previously underreported association, providing new insight into choroidal responsiveness following aflibercept therapy in treatment-naïve nAMD patients.

Numerous investigations have assessed correlations among qualitative features of neovascular complexes. In contrast, our study revealed a distinct quantitative correlation between the MNVA and choroidal structural changes, reinforcing the value of incorporating MNVA measurements into multimodal prognostic assessments. In the following section, we discuss how these findings align with, complement, or diverge from the literature evaluating neovascular membrane characteristics as predictive biomarkers in nAMD.

These observations can be contextualized within the broader literature evaluating choroidal and neovascular structural biomarkers in AMD. Toto et al. [26] demonstrated that different MNV subtypes exhibit distinct choroidal and vascular profiles, reporting a significant negative correlation between the choroidal vascularity index (CVI) and MNV flow area and a positive correlation between the CVI and choroidal thickness. These findings highlight the interplay between neovascular activity and choroidal structural integrity across MNV phenotypes. In addition, Invernizzi et al. [27] reported that choroidal thickness and the CVI increase during episodes of active neovascularization, suggesting that choroidal remodeling is dynamic and closely tied to neovascular activity. Moreover, a systematic review and meta-analysis by Hoven et al. [28] confirmed that anti-VEGF treatment consistently induces choroidal thinning during the loading phase, particularly with aflibercept; however, they did not explore SS-OCTA-based neovascular metrics as predictors of the choroidal structural response. Collectively, these studies provide important complementary evidence but also underscore that the specific quantitative relationship identified here (linking MNVA Pre to CCT Post) could represent a novel contribution to the understanding of choroidal responsiveness in nAMD patients.

Several recent studies have reinforced the prognostic relevance of structural and morphofunctional characteristics of MNV assessed by OCTA, providing an important framework for interpreting our findings. Mastropasqua et al. [29] demonstrated that quantitative measures of the MNV architecture, including the area, total vessel length, number of junctions, and fractal dimension, are significantly associated with anti-VEGF treatment response, indicating that larger and more complex lesions tend to exhibit greater therapeutic resistance. Complementing these observations, Zhang et al. [30] employed machine learning algorithms and reported that structural MNV metrics (fractal dimension, vessel area, total vessel length, and total number of end points) rank among the most influential predictors of treatment response, reinforcing that posttreatment behavior largely reflects baseline morphoangiographic complexity. In addition, Jia et al. [31] demonstrated that early changes in specific vascular parameters, particularly reductions in the greatest vascular caliber (GVC), represent independent biomarkers of clinical response at six months, whereas the baseline MNV size alone was not predictive. Taken together, these studies corroborate that quantitative OCTA-derived parameters may play crucial roles in prognostic stratification and contextualize our novel observation of a correlation between the MNVA Pre and CCT Post within a growing body of evidence linking baseline neovascular burden to subsequent structural remodeling.

### Deep capillary plexus biomarkers: AADP

Several significant associations between AADP metrics and CMT were identified, underscoring the relevance of these OCTA-derived biomarkers in characterizing both baseline exudative burden and the magnitude of anatomical recovery. First, AADP Pre demonstrated a moderate positive correlation with CMT Pre (*r* = 0.592; *p* = 0.005), indicating that greater impairment of the deep vascular plexus was associated with increased retinal thickening at presentation (item 8). This new finding expands the current evidence regarding the role of DCP alterations in treatment-naïve nAMD. The relevance of deep retinal microvascular impairment in nAMD is supported by the literature. Gao et al. [32] demonstrated that the eyes of treatment-naïve nAMD patients exhibit significantly enlarged avascular areas within the DCP when assessed with projection-resolved OCTA, indicating substantial microvascular rarefaction at this level. These findings support that deep plexus nonperfusion represents a structural manifestation of retinal microvascular compromise in patients with nAMD. In this context, the moderate positive correlation between the AADP Pre and CMT Pre in our study expands upon prior evidence, suggesting that deep capillary plexus rarefaction may serve as a clinically meaningful surrogate of initial exudative burden.

Second, AADP Pre showed a moderate negative correlation with the absolute CMT Reduction after the loading phase (*r* = − 0.581; *p* = 0.006), suggesting that higher initial AADP values may be linked to a greater structural response to treatment (item 9). Third, the percentage reduction in the AADP was moderately positively correlated with the CMT Pre (*r* = 0.674; *p* = 0.001), implying that eyes with greater initial thickening tended to exhibit greater relative improvements in the AADP over time (item 10). Finally, the percentage reduction in AADP demonstrated a moderate positive correlation with the percentage reduction in the CMT (*r* = 0.558; *p* = 0.009), indicating that changes in deep plexus metrics paralleled the proportional resolution of the retinal edema (item 11). Together, these findings indicate that the AADP captures both initial exudative load and therapeutic dynamics, supporting its role as a potential complementary OCTA-derived biomarker. Clinically, these correlations suggest that a greater AADP Pre may help in identifying eyes with more severe presentations and potentially more robust anatomical recovery. Moreover, reductions in the AADP mirroring those in the CMT reinforce its potential utility as a surrogate marker of treatment efficacy. The incorporation of the AADP into routine evaluations may therefore increase prognostic accuracy and support more individualized management strategies for nAMD.

The associations reported herein expand the current literature on nAMD biomarkers. While previous investigations have explored correlations between OCT- and OCTA-derived neovascular lesion characteristics and retinal thickness parameters, the relationships between DCP avascular metrics and both the magnitude and proportionality of structural recovery after aflibercept therapy remain incompletely characterized. Consequently, the observed correlations, linking the AADP Pre to the subsequent reduction in the CMT, as well as the proportional improvements in the AADP to both the baseline exudative burden and percentage CMT reduction, introduce previously unreported functional and structural interactions. These results broaden the spectrum of SS-OCTA biomarkers and may be relevant for assessing exudative activity, while also representing potential biomarkers that could help predict treatment responses in patients with nAMD.

### Deep plexus and choroidal changes

A low but statistically significant positive correlation between the percentage reduction in the AADP and the percentage reduction in the CCT was observed (*r* = 0.447; *p* = 0.042), indicating that eyes with greater improvement in deep plexus alterations also tended to show proportionally greater choroidal thinning after treatment (item 12). This relationship suggests a modest but measurable interplay between microvascular reorganization and the choroidal structural response during loading. Clinically, these findings highlight that simultaneous evaluation of the AADP and CCT may provide additional insight into the multidimensional effects of therapies on the retinal and choroidal compartments. Although the strength of the correlation is low, its statistical significance underscores the potential relevance of combined SS-OCT and SS-OCTA biomarkers in characterizing treatment responsiveness, reinforcing the value of integrating microvascular and choroidal parameters into comprehensive disease monitoring. To our knowledge, no published study has reported a statistically significant correlation between a reduction in the AADP and a decrease in the CCT in treatment-naïve eyes of nAMD patients treated with aflibercept. As a result, this correlation provides additional evidence of a previously underexplored interaction between DCP perfusion and choroidal remodeling during the loading phase. These findings expand the current framework of SS-OCTA-derived biomarkers and provide new insights into the multilevel vascular responses elicited by aflibercept therapy in nAMD patients.

### Superficial plexus biomarkers: AASP

A moderate negative correlation between the percentage reduction in the AASP and the absolute reduction in the CCT was also observed (item 13) (*r* = − 0.582; *p* = 0.006). The percentage reduction in the AASP demonstrated a low but significant positive correlation with the percentage reduction in the CCT (item 14) (*r* = 0.440; *p* = 0.046). Both correlations showed that the reduction in the AASP was associated with the reduction in the CCT. In practical terms, eyes exhibiting more substantial regression of superficial plexus abnormalities also tended to experience more pronounced choroidal thinning following the loading phase. Clinically, these findings suggest that recovery of the superficial vascular plexus may be linked to deeper choroidal structural remodeling, reinforcing that SS-OCTA-derived microvascular biomarkers can reflect not only retinal changes but also the broader choroidal response to aflibercept therapy in patients with nAMD. No published studies have evaluated or demonstrated a statistically significant correlation between a reduction in the AASP and a decrease in the CCT in the treatment-naïve eyes of patients with nAMD receiving a loading dose. This association adds novel quantitative evidence to the SS-OCTA literature regarding the relationship between superficial plexus alterations and choroidal remodeling during aflibercept treatment in nAMD.

Taken together, the significant correlations identified across structural, functional, and vascular biomarkers demonstrate that nAMD exhibits highly interdependent anatomical and microvascular profiles both at baseline and throughout the therapeutic course. The consistent relationships observed, such as those linking the CMT Pre to its own magnitude of reduction, the CCT Pre to the CCT Post, the BCVA to the CMT at multiple timepoints, and SS-OCTA-derived biomarkers (MNVA, AADP and AASP) to both retinal and choroidal responses, indicate that several parameters measured at presentation not only reflect the initial disease severity but are also significantly associated with the subsequent anatomical trajectory during aflibercept therapy. Clinically, this integration of multimodal biomarkers supports a more refined interpretation of disease behavior and treatment-related anatomical changes, with potential implications for individualized monitoring strategies. These findings underscore the relevance of incorporating a comprehensive structural and vascular biomarker panel into routine evaluations, as the inclusion of multiple biomarkers enhances a clinician’s ability to characterize disease burden, estimate therapeutic benefit, and ultimately optimize management of nAMD.

Although 4 of the 14 correlations identified in our analysis were classified as low in magnitude, these findings should be interpreted with caution rather than dismissed. All of these associations were statistically significant and demonstrated biologically plausible directional trends. It is plausible that the relatively small sample size of our cohort attenuated the observed correlation strengths, suggesting that larger studies may reveal more robust relationships. As highlighted by Mukaka [15], correlation coefficients should always be interpreted within their appropriate clinical and biological context, and even weak associations may yield meaningful insights when they are systematic and biologically plausible. Moreover, the classification of correlation strength is not universal and depends on the interpretative thresholds adopted, which are often presented as guideline-based rather than absolute criteria, as discussed by Chan [33]. In our study, we adopted a widely accepted framework for interpreting correlation coefficients, thereby ensuring methodological robustness, reproducibility, and comparability with existing literature. To our knowledge, this represents the first prospective investigation to systematically evaluate correlations between SS-OCT and SS-OCTA biomarkers in treatment-naïve eyes with nAMD undergoing a standardized aflibercept loading regimen, underscoring the originality and potential clinical relevance of these findings.

### Limitations

This study has some limitations that should be considered: a-) Its single-center design. b-) Relatively small sample size may restrict the generalizability of the findings and limit statistical power. Although the present study included a relatively small sample size, several design features strengthen its internal validity, including its prospective nature, standardized imaging acquisition, uniform anti-VEGF treatment protocol, and the inclusion of treatment-naïve eyes only. These methodological aspects help reduce measurement variability and selection bias. Nevertheless, the limited sample size may decrease the statistical power to detect weaker associations between biomarkers, and therefore the findings should be interpreted cautiously as hypothesis-generating and warrant confirmation in larger prospective studies. c-) Although SS-OCT angiography enables high-resolution visualization of the retinal and choroidal microvasculature, quantitative metrics derived from SS-OCTA remain susceptible to segmentation inaccuracies and motion artifacts, which may introduce some degree of measurement variability, despite careful image acquisition, manual verification, and masked grading. d-) Present analyses were exploratory in nature and relied on bivariate correlation models, consistent with the study objective of characterizing biomarker interrelationships rather than establishing independent predictive effects. Correlations between baseline values with its own change score (Δ = post − pre) should be interpreted with caution, as they may be influenced by mathematical coupling and regression to the mean, and therefore do not imply predictive or causal relationships. Future studies employing multivariable or partial correlation approaches may further clarify the independence of these associations. e-) The 4-month follow-up period constrains the ability to determine whether these relationships are sustained over longer timeframes; extended longitudinal follow-up would be valuable for assessing the temporal stability of these biomarkers, although the primary objective of this study was to evaluate short-term changes over a 4-month interval. f-) Finally, another limitation of this study is that only patients with type 1 MNV were included. This approach was intentionally adopted to maintain anatomical and pathophysiological homogeneity when evaluating correlations between structural and vascular biomarkers using SS-OCT and SS-OCTA. However, different subtypes of MNV (type 2 and type 3) present distinct anatomical locations, vascular architectures, and biological behaviors, which may influence imaging biomarkers and treatment response. Therefore, the findings of this study may not be directly generalizable to AMD with type 2 and 3 MNV. Future studies including multiple MNV subtypes are needed to determine if the observed relationships between biomarkers are consistent across different neovascular phenotypes.

Despite these limitations, the present prospective assessment offers novel and clinically relevant insights into the anatomical and microvascular response to aflibercept in treatment-naïve nAMD patients based on comprehensive SS-OCT and SS-OCTA biomarker evaluation.

From a clinical perspective, the correlations identified in this study suggest that multimodal biomarker assessment using SS-OCT and SS-OCTA may assist clinicians in better characterizing disease severity and early treatment response in treatment-naïve nAMD. Baseline structural parameters such as CMT and CCT, together with vascular metrics including MNVA, AADP, and AASP, may provide complementary information regarding the anatomical burden of disease and the magnitude of structural remodeling during the loading phase of anti-VEGF therapy. Although these correlations should not be interpreted as predictive models, they highlight the potential value of integrating structural and vascular biomarkers when monitoring therapeutic response and may contribute to more individualized clinical assessment of patients with nAMD.

## Conclusion

Our prospective investigation revealed that treatment-naïve nAMD eyes undergo coordinated remodeling of functional, structural, and microvascular compartments in response to aflibercept loading therapy. Significant improvements were observed across the SS-OCT and SS-OCTA biomarkers, accompanied by multiple statistically significant correlations linking baseline disease burden to posttreatment anatomical and vascular outcomes. These findings represent previously unreported correlations in the literature, providing novel insights into aflibercept therapy and expanding the current understanding of structural and vascular responses in treatment-naïve nAMD. The baseline CMT and CCT emerged as candidate biomarkers associated with posttreatment retinal and choroidal status. In addition, SS-OCTA-derived metrics (MNVA, AADP, and AASP) were closely related to both retinal thickness and choroidal remodeling, highlighting previously unrecognized interactions between neovascular burden and the microvascular choroidal response. Notably, the inverse association between MNVA Pre and CCT Post indicates that greater neovascular burden is associated with more pronounced choroidal thinning after VEGF suppression. Overall, these findings show that nAMD involves coordinated retinal, choroidal, and microvascular changes during early aflibercept therapy. Combining SS-OCT and SS-OCTA biomarkers provides a clearer view of disease behavior and treatment response, supporting a more accurate prognosis and a more personalized approach to patient management.

## Data Availability

All datasets presented in the study are included in the article/Supplementary Material.

## Abbreviations

AADP: Avascular area of the deep plexus
AMD: Age-related macular degeneration
anti-VEGF: Anti-vascular endothelial growth factor
AASP: Avascular area of the superficial plexus
BCVA: Best-corrected visual acuity
CAAE: Certificado de Apresentação de Apreciação Ética / Ethics Committee Identifier (Brazil)
CCT: Central choroidal thickness
CMT: Central macular thickness
CRT: Central retinal thickness
CST: Central subfield thickness
CVI: Choroidal vascularity index
DCP: Deep capillary plexuses
ETDRS: Early treatment diabetic retinopathy study
FAZ: Foveal avascular zone
GVC: Greatest vascular caliber
IOP: Intraocular pressure
IRF: Intraretinal fluid
logMAR: Logarithm of the minimum angle of resolution
MNVA: Macular neovascularization area
MNV: Macular neovascularization
nAMD: Neovascular age-related macular degeneration
OCT: Optical coherence tomography
OCTA: Optical coherence tomography angiography
PED: Pigment epithelial detachment
r: Correlation coefficient
RPE: Retinal pigment epithelium
SCP: Superficial capillary plexuses
SRF: Subretinal fluid
SS-OCT: Swept-source optical coherence tomography
SS-OCTA: Swept-source optical coherence tomography angiography
VA: Visual acuity
VD: Vessel density
3D: Three-dimensional
